# Transcriptomic Profiles Carry Most of the Multi-Omics Signal for Drug Sensitivity Prediction in Cancer Cell Lines: A Cross-Lineage and Independent-Screen Evaluation of Twelve Anticancer Agents

**DOI:** 10.3390/cancers18213503

**Published:** 2026-10-30

**Authors:** Sultan A. Alfawaz, Haifa Almukadi, Thoraia Shinawi, Dalal Alfawaz, Dareen Alyousfi, Khalda K. Nasser, Ashraf A. El Harouni, Noor A. Shaik, Babajan Banaganapalli

**Affiliations:** 1 Department of Clinical Pharmacology, Faculty of Medicine, King Abdulaziz University, Jeddah 21589, Saudi Arabia; 2 Department of Pharmacology and Toxicology, Faculty of Pharmacy, King Abdulaziz University, Jeddah 21589, Saudi Arabia; 3 Department of Medical Laboratory Sciences, Faculty of Applied Medical Sciences, King Abdulaziz University, Jeddah 21589, Saudi Arabia; 4 Department of Clinical Biochemistry, Faculty of Medicine, King Abdulaziz University, Jeddah 21589, Saudi Arabia; 5 Department of Genetic Medicine, Faculty of Medicine, King Abdulaziz University, Jeddah 21589, Saudi Arabia; 6 Princess Al-Jawhara Al-Ibrahim Center of Excellence in Research of Hereditary Disorders, King Abdulaziz University, Jeddah 21589, Saudi Arabia; 7 Center of Excellence in High Performance Computing (Aziz), King Abdulaziz University, Jeddah 21589, Saudi Arabia

**Keywords:** drug sensitivity prediction, multi-omics integration, pharmacogenomics, machine learning, cross-lineage validation, external validation, SHAP, DepMap, GDSC, PRISM

## Abstract

**Background/Objectives:** Combining omics layers is widely expected to improve cancer drug response prediction, but this is rarely tested under family-wise error control or on an independent screen. We measured what somatic mutation and copy number data add to gene expression, and how well models transfer to unseen lineages and an external screen. **Methods:** We linked the Cancer Dependency Map transcriptomic, somatic mutation and copy number profiles to GDSC2 dose–response data for twelve mechanistically diverse anticancer compounds in 509 cell lines. Lineage-only, single-modality, early-fusion and stacked late-fusion gradient-boosting models were compared by repeated cross-validation (DeLong tests, Holm adjustment), leave-one-lineage-out transfer across eleven solid tumour histologies, and external validation on 77-163 held-out PRISM cell lines per compound. **Results:** Gene expression models outperformed tissue-of-origin baselines for all twelve agents (AUC 0.69–0.84; gains 0.024–0.169; Holm-adjusted *p* < 0.001 for seven drugs). Adding mutation and copy number data changed AUC by −0.015 to +0.035, and no per-drug gain survived multiplicity correction. Pooled across compounds, early fusion added 0.008 AUC (95% CI 0.000–0.016), a gain that fold-level re-analysis did not support. Mixed-lineage cross-validation exceeded leave-one-lineage-out AUC by a median of 0.030 (*p* = 0.0005). External PRISM AUCs ranged from 0.46 to 0.76 and followed the concordance between the two screens (Spearman rho = 0.72, *p* = 0.008). TreeSHAP attributions recovered known determinants, including *SLFN11*, *TP53*, *BRAF*, *PIK3CA* and downstream MAPK transcriptional effectors. **Conclusions:** In this twelve-drug panel, gene expression captured most of the predictable response variation. Accuracy fell on held-out lineages and on an independent screen, which sets empirical limits on how far these models can generalise.

## Simple Summary

Computer models that predict how cancers respond to drugs are usually trained on laboratory cancer cell lines tested against many compounds. Combining several types of molecular data, such as gene mutations, copy number changes and gene activity, is often expected to predict better than gene activity alone. We tested this for twelve anticancer drugs on more than five hundred cell lines. Gene activity profiles captured nearly all of the response differences that could be predicted. Adding mutation and copy number data raised accuracy by only about 0.01 in the area under the ROC curve on average, and no single drug kept a confirmed benefit after statistical correction. Predictions were much less accurate for cancer types left out of the training and for an independent drug screen, where accuracy depended on how closely the two screens agreed. These results apply to the drugs, screens and tree-based models studied here.

## 1. Introduction

Tailoring therapeutic regimens to the molecular profile of an individual tumour remains a central aspiration of precision oncology, yet anticipating whether a patient will benefit is difficult for most systemic treatments [1,2]. Although targeted therapies produce meaningful clinical responses in patient subsets harbouring actionable alterations, basket trials enrolling patients solely on the basis of driver mutations frequently observe that drug activity varies substantially across histologies [2,3]. Machine-learning models designed to predict therapeutic response from high-dimensional molecular profiles have therefore become central to precision oncology strategies.

High-throughput pharmacogenomic screens provide the empirical bedrock for training and evaluating such models. The Genomics of Drug Sensitivity in Cancer (GDSC) consortium has generated dose–response curves for hundreds of agents across approximately one thousand cancer cell lines [4,5], while the *Cancer Cell Line Encyclopaedia*, now consolidated within the Cancer Dependency Map (DepMap), supplies matched transcriptomic, mutational, and copy number data for these models [6,7,8]. Early crowdsourced competitions and subsequent investigations identified baseline gene expression as the most informative single data modality for predicting drug response [9,10]. Motivated by these findings, diverse deep-learning and multimodal architectures combining multiple genomic layers have been actively proposed [11,12].

Evaluating the actual utility of these multi-omics models is hampered by three recurrent methodological challenges. Most reported fusion gains rely on unadjusted point estimates from a single cohort, leaving it ambiguous whether minor AUC increments reflect genuine biological complementarity or mere sampling fluctuation. In addition, conventional cross-validation designs that pool distinct lineages across folds allow models to exploit tissue-specific markers correlated with baseline sensitivity; this pooling frequently conceals sharp performance declines when models are tasked with predicting the response in cancer types omitted from training [13,14]. Finally, independent pharmacogenomic screens often exhibit only modest concordance for identical compounds [15]. When a model performs poorly on external cell lines, determining whether the failure reflects fragile learned associations or divergence between screening assays is rarely straightforward a distinction that large secondary viability datasets like PRISM [16] allow investigators to dissect directly.

Here, we benchmark multi-omics data integration directly against transcriptomic and tissue-of-origin baselines across twelve anticancer agents spanning distinct mechanisms of action, strictly embedding all data-dependent preprocessing inside training folds to prevent data leakage [14]. Across 509 well-characterised cell lines, we measure how discrimination shifts when somatic alterations and copy numbers are added to gene expression, quantify performance attrition under leave-one-lineage-out validation, and evaluate generalisation to held-out PRISM profiles. We reason that multi-omics fusion would provide minimal benefit for broadly active cytotoxic drugs but yield measurable advantages for targeted agents dependent on discrete oncogenic drivers. Lastly, we evaluate whether TreeSHAP feature attribution reliably recovers established pharmacogenomic biomarkers.

## 2. Materials and Methods

### 2.1. Study Design and Reporting

**Figure 1:**
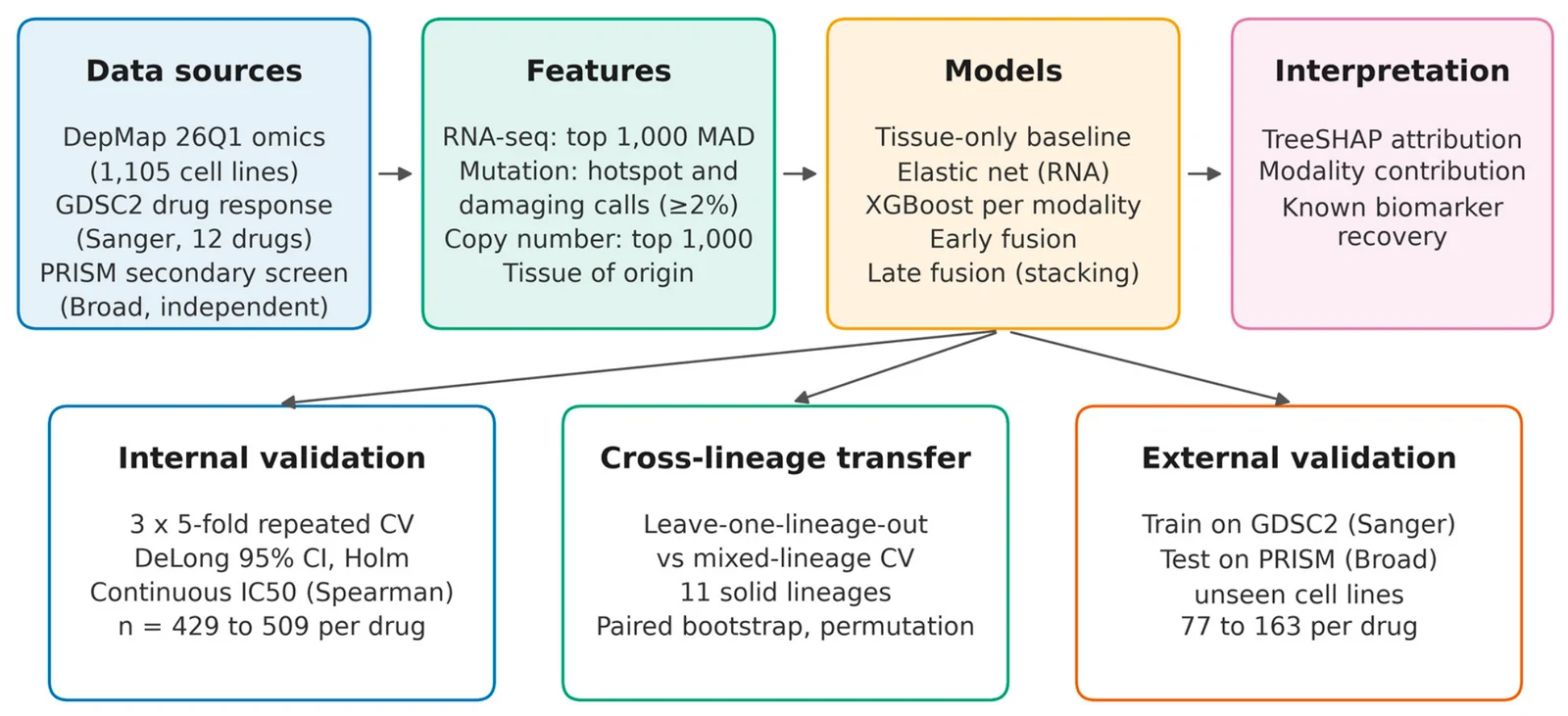
Study workflow. Matched RNA sequencing, somatic mutation and copy number profiles from DepMap were linked to GDSC2 drug response for twelve agents. Feature selection and scaling were fitted inside each training fold. Tissue-only, single-omics, early-fusion and late-fusion models were evaluated by repeated cross-validation, by leave-one-lineage-out transfer with a mixed-lineage comparator and a permutation control, and by external testing on unseen PRISM cell lines; the early-fusion model was interpreted with TreeSHAP.

This retrospective prediction modelling study utilised publicly available cancer cell line data and is reported following the TRIPOD + AI recommendations applicable to preclinical models [17]. Drug selection, feature processing, model hyperparameters, comparison families, and validation schemes were established in an internal analysis plan prior to inspecting model outputs (the plan was not publicly registered). All data-dependent preprocessing operations, including feature selection, scaling, and imputation, were fitted strictly within each training fold to avoid information leakage [14]. Sensitivity analyses conducted during peer review are detailed in Section 2.11 and presented separately from the primary analyses. Figure 1 depicts the overall study workflow.

### 2.2. Data Sources

Omics and annotation data were obtained from the DepMap 26Q1 release: model annotations (Model.csv), protein-coding gene expression as log2(TPM + 1) (19,215 genes), somatic mutations and gene-level relative copy number derived from whole-genome sequencing (19,366 genes) [7,8]. Only the default sequencing entry for each model was kept. Drug response was taken from the GDSC2 fitted dose–response release of 27 October 2023, distributed through the Cell Model Passports resource, and linked to DepMap through the Sanger model identifier [4,5]. External response data came from the PRISM Repurposing secondary screen (release 20Q2) and was restricted to cell lines that passed short tandem repeat profiling [16]. In total, 1105 models had all three omics layers, and 509 of these had GDSC2 measurements for at least one study drug.

### 2.3. Drugs and Response Definition

Twelve compounds were selected to represent diverse mechanisms of action and because each had been profiled across both GDSC2 and PRISM: six cytotoxic agents (docetaxel, paclitaxel, cisplatin, gemcitabine, 5-fluorouracil, and irinotecan) and six targeted inhibitors (the MDM2 antagonist nutlin-3a, the BRAF inhibitor dabrafenib, the MEK inhibitor trametinib, the dual EGFR/ERBB2 inhibitor lapatinib, the EGFR inhibitor erlotinib, and the PI3Kα inhibitor alpelisib) (Table 1). Replicate measurements of the natural log of the half-maximal inhibitory concentration (LN_IC50) were averaged per cell line. For binary classification, lines with LN_IC50 below the drug-specific median were designated sensitive, providing balanced class distributions. For continuous regression, the target was defined as -LN_IC50, such that higher values denote greater sensitivity. This median split represents an operational statistical threshold to standardise AUC comparisons across agents rather than a clinically validated boundary of drug addiction particularly for targeted drugs where only a minority of cell lines are oncogene-dependent. To evaluate the sensitivity of our findings to this threshold, two alternative binarisation cut-offs and continuous regression models were analysed in parallel (Section 2.11).

### 2.4. Feature Processing

Within each training fold, the 1000 expressed genes exhibiting the highest median absolute deviation were selected and standardised. Copy number features lacking observed values were discarded, the remaining missing entries were imputed with the training-fold median, and the 1000 genes with the highest variance were retained and standardised. Mutations were encoded per gene as two binary indicators: one indicating hotspot or oncogenic high-impact variants, and the other denoting damaging alterations (probable loss of function or high-impact tumour-suppressor mutations). Indicators present in at least 2% of training lines were retained (yielding roughly 350 to 470 features per drug). In the evaluated cohort, 753 of 19,366 genes lacked copy number measurements across all lines and were omitted; because no missing values occurred among the remaining genes, median imputation did not alter any observed data.

### 2.5. Models

In this project, we trained seven distinct models per drug. A tissue-only baseline using lineage indicators served as the reference standard against which molecular models were compared. Single-modality classifiers were trained independently on expression (RNA), mutation (MUT), and copy number (CN) feature blocks. Early fusion concatenated the three processed matrices into a unified feature set. Late fusion employed stacked generalisation [18]: within each training fold, an internal five-fold split produced out-of-fold predictions from the three single-modality models; a logistic regression meta-learner was fitted on these predictions; and the meta-learner was then evaluated on outer test folds. All tree-based models used XGBoost [19] with fixed hyperparameters (300 estimators, maximum depth 3, learning rate 0.05, subsample ratio 0.8, feature subsample ratio 0.5, minimum child weight 3) specified a priori without tuning, avoiding optimistic selection bias. An elastic-net logistic regression [20] (mixing ratio 0.5, inverse regularisation parameter 0.05) served as a linear comparator for expression. For continuous prediction, XGBoost regressors with identical parameters were trained on expression and early-fusion features.

### 2.6. Internal Validation and Statistical Comparison

Internal performance was assessed via stratified five-fold cross-validation repeated three times with independent random splits. Out-of-fold probabilities were averaged across repeats for each line, and the area under the receiver operating characteristic curve (AUC) with 95% confidence intervals (CIs) was calculated using the DeLong method [21]; cross-repeat standard deviations are likewise reported. Paired AUC differences were tested using DeLong’s test across five pre-specified comparison families (early fusion versus RNA, late fusion versus RNA, RNA versus tissue, XGBoost versus elastic net for RNA, and mutation versus tissue), with *p*-values adjusted via the Holm procedure across the twelve drugs in each family [22]. Because repeated cross-validation partitions share considerable training data, standard DeLong testing treats ensembled predictions as fixed, capturing finite-sample testing uncertainty but neglecting the variance induced by retraining across resampled cohorts. We addressed this potential anti-conservatism through two complementary approaches: evaluating DeLong tests separately within each individual split, and re-running cross-validation under newly initialised partitions with complete fold-level tracking to evaluate the 15 paired fold-level AUC differences with the Nadeau-Bengio corrected resampled *t*-test [23]. Overall fusion effects across the 12 drugs were synthesised using a DerSimonian–Laird random-effects model with DeLong standard errors [24], alongside Wilcoxon signed-rank tests of paired differences across compounds. Because non-significant differences cannot demonstrate equivalence, we determined whether each drug’s 90% CI fell entirely within a practical equivalence window of +/− 0.03 AUC [25], and calculated the minimum effect size detectable at 80% power under uncorrected (alpha = 0.05) and Holm-adjusted (alpha = 0.05/12) thresholds. Differences in fusion gains between targeted and cytotoxic agents were tested with two-sided Mann–Whitney U tests. Continuous predictions were evaluated by Spearman’s rank correlation between observed and predicted values (with 95% CIs derived from 1000 bootstrap iterations); correlation shifts from early fusion were tested using 2000 paired bootstrap draws under Holm control. Significance was defined at two-sided alpha = 0.05.

### 2.7. Cross-Lineage Transfer

Cross-lineage analyses were restricted to solid lineages comprising at least 15 cell lines for a given compound, yielding eleven lineages (lung, breast, oesophagus and stomach, skin, central nervous system, bowel, ovary and fallopian tube, pancreas, bone, kidney, and head and neck) and 130 drug-lineage combinations; haematological lineages were excluded from evaluation because only two were available (lymphoid, *n* = 50; myeloid, *n* = 19) and they differ from solid tumours in biology and growth in culture, so the transfer conclusions apply to solid tumours. In both schemes, training sets contained every other cell line screened with the drug, including haematological and smaller solid lineages. Because baseline sensitivity varies by tissue, classification labels were centred on the median LN_IC50 within each lineage, preventing models from exploiting simple tissue recognition. In leave-one-lineage-out (LOCO) validation, models were trained on all other histologies and tested on the withheld lineage. As a matched comparator, mixed-lineage cross-validation (five folds, two repeats, stratified by lineage and label) was executed across the full panel, and its predictions for the identical withheld lines were extracted to ensure both paradigms were evaluated on identical cell lines with comparable training volumes. Paired differences (mixed minus LOCO) were assessed using 2000 bootstrap iterations per pair and across all pairs via Wilcoxon signed-rank tests. Bootstrap 95% CIs were computed for every LOCO estimate, and the comparison was repeated with minimum lineage sizes of 20, 25 and 30 cell lines to examine the effect of the 15-line threshold. Within-lineage cross-validation was excluded as a benchmark because small cross-validated samples produce null AUCs systematically depressed below 0.5 [26], an artefact confirmed here by a permutation control repeating within-lineage validation across 20 label-permuted iterations per drug-lineage pair (2600 runs).

### 2.8. External Validation

For each drug, expression, early-fusion, late-fusion and tissue-only models were trained on the complete GDSC2 cohort and applied to PRISM cell lines that had DepMap omics data but were absent from the GDSC2 training set for that drug. The PRISM area-under-the-dose–response-curve values were averaged per cell line, and lines below the PRISM median of the external evaluation cohort were labelled sensitive. This threshold uses the distribution of PRISM responses in the evaluation cohort but no model predictions, and AUC depends only on how predictions rank cell lines. A prospective user would not know that distribution, so the analysis was repeated with two thresholds fixed without evaluation-cohort data: the median PRISM AUC of the cell lines screened in both GDSC2 and PRISM, none of which were evaluated, and the median over every PRISM cell line screened with the drug. The Spearman correlation between predicted sensitivity and PRISM AUC, which needs no threshold, was also reported. Late fusion and the alternative thresholds were evaluated in a rebuild of the external analysis from the raw PRISM data that used the same protocol with a different random seed. External AUCs were reported with 95% CIs from 2000 bootstrap resamples. To quantify agreement between the two screens, the Spearman correlation between GDSC2 LN_IC50 and PRISM AUC was computed on cell lines measured in both, and its association with external AUC across drugs was tested with the Spearman correlation, with a bootstrap CI over drugs, a leave-one-drug-out analysis and a partial correlation adjusted for internal cross-validated AUC. No batch correction was applied between data sources. All omics data came from a single DepMap release, and responses from the two screens were never pooled: GDSC2 data were used only for training and PRISM data only for testing, with labels defined within each screen relative to its own distribution. This removes differences in measurement scale between screens but cannot remove differences in the phenotype each assay measures.

### 2.9. Model Interpretation

For each drug, an early-fusion model was trained on the full cohort and explained with TreeSHAP [27,28]. Features were ranked by the mean absolute SHAP value, attribution shares were summed by modality, and the direction of each feature’s effect was summarised as the correlation between its value and its SHAP value. Known biomarkers with established mechanistic links to the study drugs were selected from the literature, and their ranks were extracted.

### 2.10. Software

Analyses were implemented in Python 3.13.12 using XGBoost 3.2.0, scikit-learn 1.8.0 [29], SciPy 1.17.1, NumPy 2.4.5, pandas 2.3.3 and Matplotlib 3.10.9; TreeSHAP feature attributions were calculated with the SHAP 0.51.0 package. All random seeds were fixed so that the results can be reproduced exactly. Revision analyses used pandas 3.0.6, NumPy 2.5.4, SciPy 1.18.1, scikit-learn 1.8.0 and XGBoost 3.2.0, and the code is available as described in the Data Availability Statement.

### 2.11. Sensitivity Analyses

Three sensitivity analyses used the cross-validation re-run described in Section 2.6. First, to test whether the dominance of expression depended on the unsupervised variance filter, six drug-specific biomarkers per drug, chosen from the mechanism of action (for example *ABCB1*, *ABCG2* and *TUBB3* for the taxanes, and *ERBB2*, *EGFR* and *GRB7* for lapatinib; Table S20), were added to the expression features whenever the filter did not select them, together with the copy number of relevant amplified drivers such as *ERBB2*, *EGFR* and *MDM2*. The augmented expression and early-fusion models were compared with their unaugmented versions. Second, response was redefined in two ways: the most sensitive third of cell lines was compared with the most resistant third after removing the middle third, and only the most sensitive quarter was labelled sensitive, which is closer to the minority of dependent lines expected for targeted agents. Third, a subtype baseline replaced the 26 lineage indicators with indicators for the 58 OncoTree primary diseases in the cohort [30], which separate, for example, non-small cell lung cancer from lung neuroendocrine tumours and oesophageal squamous cell carcinoma from oesophagogastric adenocarcinoma.

## 3. Results

### 3.1. Cohort Characteristics

The analysis cohort comprised 509 cell lines from 26 lineages, most frequently lung (*n* = 99), lymphoid (*n* = 50), breast (*n* = 41), oesophagus and stomach (*n* = 34), skin (*n* = 28) and central nervous system (*n* = 28). Between 429 (cisplatin) and 509 cell lines were available per drug (Table 1). For external testing, between 77 (gemcitabine) and 163 (erlotinib and trametinib) PRISM cell lines per drug had not been seen during training. On cell lines screened in both resources, agreement between the GDSC2 and PRISM response was modest, with Spearman correlations of 0.12 for cisplatin to 0.61 for trametinib.

### 3.2. Expression Models Outperform Tissue-of-Origin Baselines

**Figure 2:**
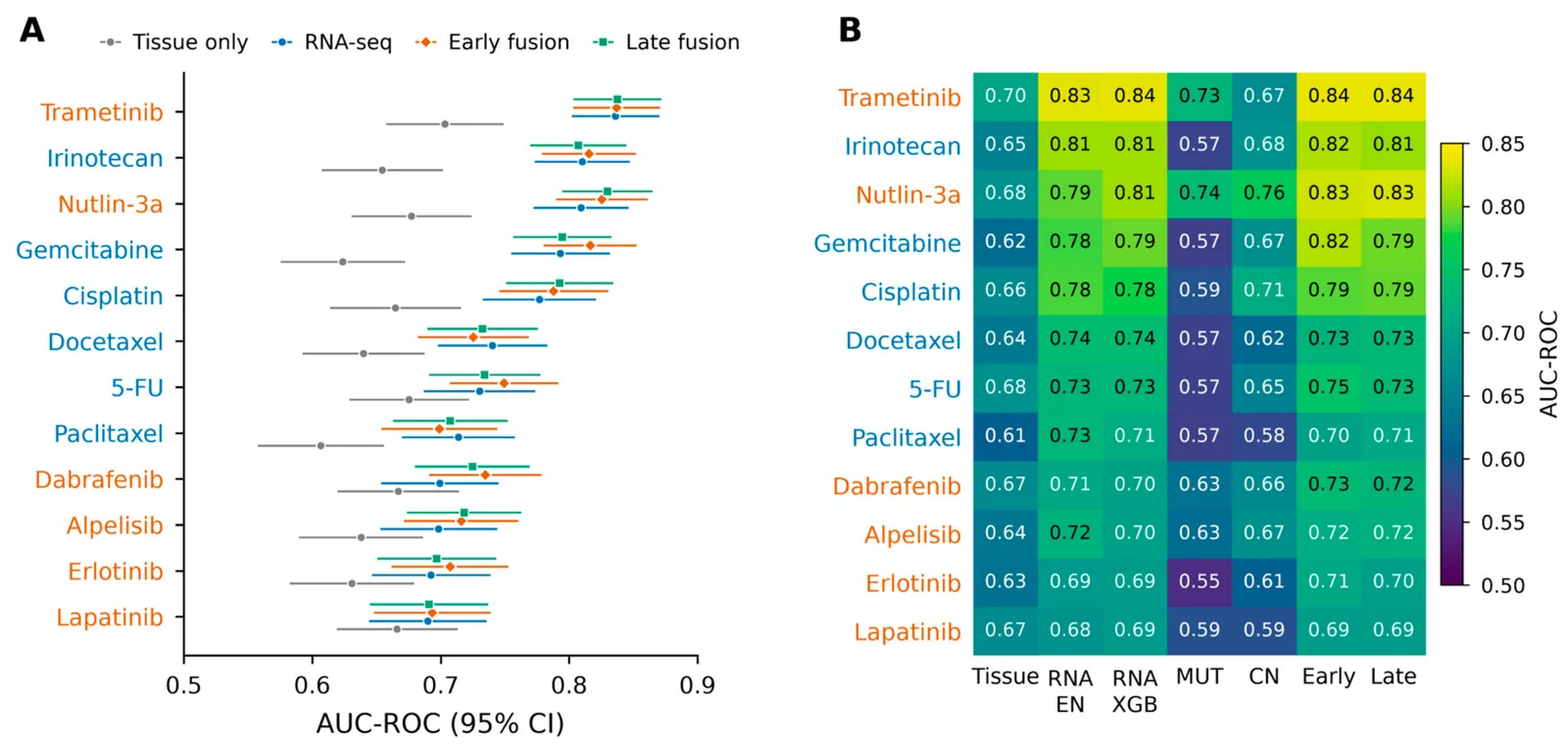
Internal cross-validated performance. (**A**) AUC with 95% DeLong confidence intervals for the tissue-only, expression (RNA-seq), early-fusion and late-fusion models for each drug, ordered by expression model AUC. (**B**) AUC for all seven models. Drug names are coloured by class (targeted, vermilion; cytotoxic, blue). EN, elastic net; XGB, XGBoost; MUT, mutation; CN, copy number. Results are from three repeats of stratified five-fold cross-validation with out-of-fold probabilities averaged across repeats.

The tissue-only baseline reached AUCs between 0.607 (paclitaxel) and 0.703 (trametinib), so lineage alone carries substantial information about drug response (Figure 2, Table S8). Expression models exceeded this baseline for all twelve drugs, with AUCs between 0.690 (lapatinib) and 0.836 (trametinib) and gains of 0.024 to 0.169. The improvement was significant after the Holm correction was performed for seven drugs: gemcitabine (+0.169), irinotecan (+0.156), trametinib (+0.133), nutlin-3a (+0.132), cisplatin (+0.112), paclitaxel (+0.107) and docetaxel (+0.100), all with adjusted *p* of 0.0003 or less. Gains for alpelisib, erlotinib and 5-fluorouracil were borderline (adjusted *p* = 0.054 to 0.063), and those for dabrafenib and lapatinib were not significant (adjusted *p* = 0.32 and 0.34). Mutation-only models were the weakest single modality (median AUC 0.581). Their AUC was numerically above the tissue baseline only for nutlin-3a (0.739) and trametinib (0.728), and neither difference was significant after correction (adjusted *p* = 0.20 and 0.91); for 5-fluorouracil, the mutation-only model was significantly worse than the tissue model (adjusted *p* = 0.009); copy number models were intermediate (median AUC 0.67). XGBoost and elastic-net expression models performed similarly, and no difference was significant after correction (largest difference 0.023 for alpelisib, adjusted *p* = 0.46). Variability across cross-validation repeats was small (standard deviation of AUC at most 0.021 for the expression model and 0.031 for the mutation-only model). For continuous sensitivity, the Spearman correlation between predicted and observed values ranged from 0.39 (alpelisib) to 0.66 (irinotecan and nutlin-3a) for the expression model (drug-wise values in Tables S6 and S14).

### 3.3. Multi-Omics Fusion Gives Small Gains That Are Not Confirmed for Individual Drugs

**Figure 3:**
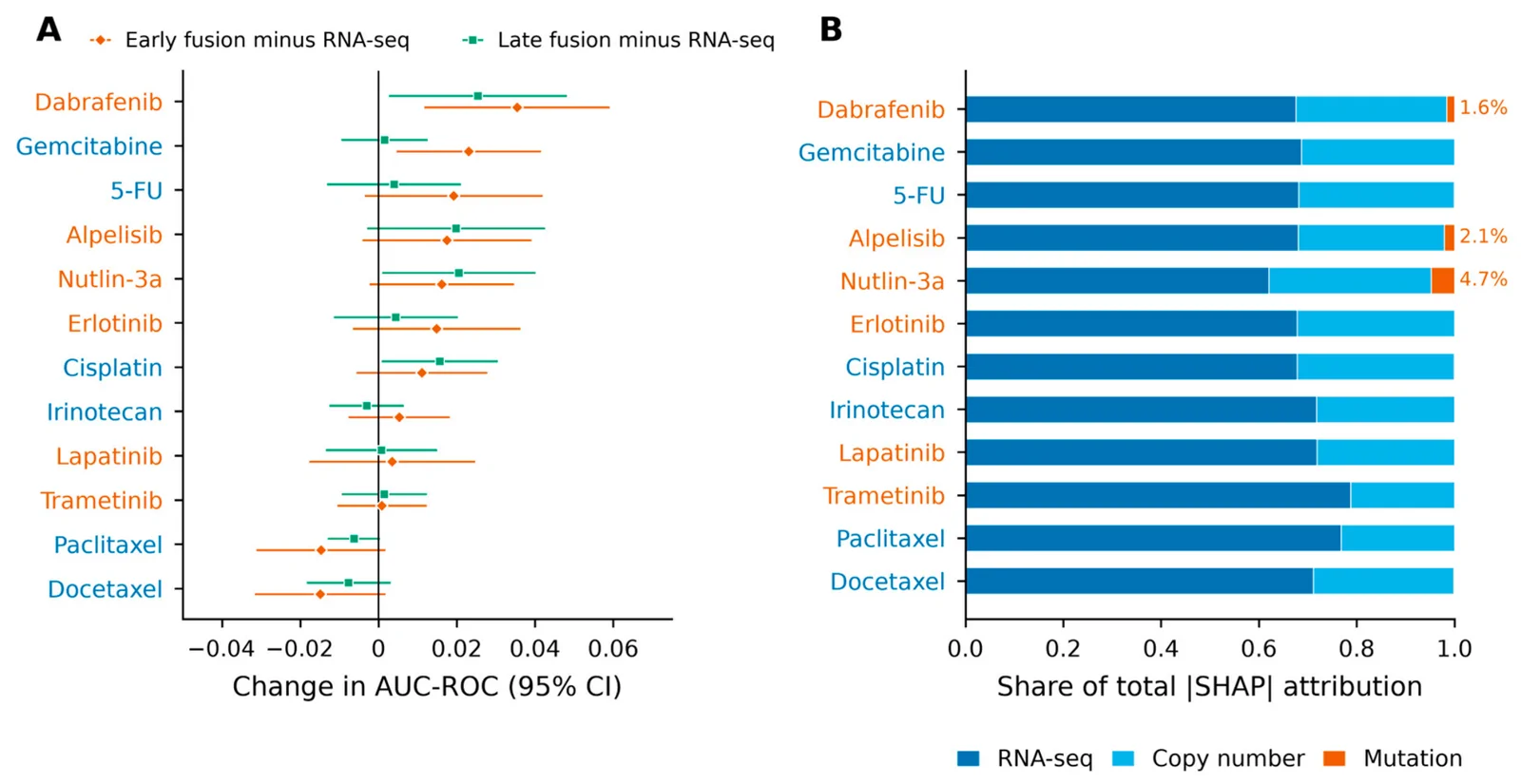
Contribution of mutation and copy number data. (**A**) Paired change in the AUC for early fusion and late fusion relative to the expression model, with 95% DeLong confidence intervals; no difference was significant after the Holm correction. (**B**) Share of the total mean absolute SHAP attribution by modality in the early-fusion model; values give the mutation share where it exceeded 1%. Drug names are coloured by class.

Adding the mutation and copy number features to expression changed the AUC by −0.015 to +0.035 with early fusion and by −0.008 to +0.025 with late fusion (Figure 3A, Table S9). The largest gain was for dabrafenib with early fusion (+0.035, 95% CI +0.012 to +0.059, *p* = 0.003), and it did not remain significant after the Holm correction (adjusted *p* = 0.081). Nominally significant late-fusion gains for dabrafenib (+0.025, *p* = 0.028), nutlin-3a (+0.021, *p* = 0.040) and cisplatin (+0.016, *p* = 0.038), and an early-fusion gain for gemcitabine (+0.023, *p* = 0.015), also lost significance after correction (adjusted *p* = 0.34 to 0.80). For both taxanes, early fusion slightly reduced AUC (−0.015 each). The median early-fusion gain was 0.016 for targeted agents and 0.008 for cytotoxic agents (Mann–Whitney *p* = 0.59), and the corresponding late-fusion gains were 0.012 and −0.001 (*p* = 0.093). The mutation-only AUC was numerically higher for targeted than for cytotoxic drugs (median 0.629 versus 0.571, *p* = 0.093). For regression, early fusion changed the Spearman correlation relative to expression by −0.025 to +0.058, again with the largest change for dabrafenib (0.502 to 0.560).

The absence of significant per-drug gains does not mean that the gains are zero. Early fusion increased the AUC in 10 of 12 drugs (Wilcoxon signed-rank *p* = 0.034), and the random-effects pooled gain was +0.008 (95% CI +0.000 to +0.016, *p* = 0.041; I2 = 59%). For late fusion the pooled gain was +0.003 (95% CI −0.002 to +0.009, *p* = 0.26). The upper 95% confidence limits of the per-drug early-fusion gains reached at most 0.059, and the per-drug 90% CIs lay within +/− 0.03 AUC for six of 12 drugs with early fusion, and nine of 12 with late fusion (Table S12). At 80% power, the smallest per-drug difference detectable at the strictest Holm threshold was 0.022 to 0.045 AUC for early fusion (median 0.035). The study could therefore detect per-drug gains of about 0.03 to 0.04 AUC but not smaller ones. The pooled analysis suggests a small average gain from early fusion, but in the cross-validation re-run described below early fusion was positive in eight of 12 drugs (median +0.008, Wilcoxon *p* = 0.20), so this average gain should be regarded as tentative. The continuous endpoint showed the same pattern: early fusion increased the Spearman correlation in 10 of 12 drugs (median change +0.020, Wilcoxon *p* = 0.027), with nominal paired-bootstrap significance for dabrafenib and alpelisib and none after the Holm correction (Table S14).

Inference that allows for the overlap between the training sets led to the same conclusions (Table S13). When the three original repeats were tested separately, fusion differences were small in every repeat, but their sign for some drugs and their *p* values varied between partitions (for dabrafenib early fusion, *p* from 0.0005 to 0.22), which shows how much a single partition can sway a test. In the re-run with new random partitions, AUCs were close to the published values (largest difference 0.021). With the corrected resampled *t*-test, no fusion gain was significant for any drug, even before correction, whereas the expression model remained better than the tissue baseline after the Holm correction for six of 12 drugs (10 of 12 with the DeLong test in the same re-run, and seven of 12 in the original analysis). The DeLong intervals in Tables S8 and S9 are therefore conditional on the training data; the corrected test is more conservative and does not change any conclusion about fusion.

### 3.4. Model Attribution Is Dominated by Expression

TreeSHAP decomposition of the early-fusion models attributed 62% to 79% of total absolute attribution to expression features, and 21% to 33% to copy number features (Figure 3B). Mutation features accounted for less than 1% of attribution for nine drugs, and for 1.6% (dabrafenib), 2.1% (alpelisib) and 4.7% (nutlin-3a) for the remaining three; these were also the three drugs with the largest late-fusion gains. The copy number share should not be read as unique predictive information. Attribution measures how much a fitted model uses a block of features, and when blocks are correlated, a model can use one in place of another. Among the 1000 most variable copy-number genes, the copy number correlated with the expression of the same gene (median Spearman rho 0.26, interquartile range 0.14 to 0.45), and copy-number-only models performed at about the level of the tissue baseline (median difference −0.011 AUC), consistent with the copy number carrying information that the expression and lineage largely already provide.

### 3.5. Mixed-Lineage Cross-Validation Overestimates Transfer to Unseen Lineages

**Figure 4:**
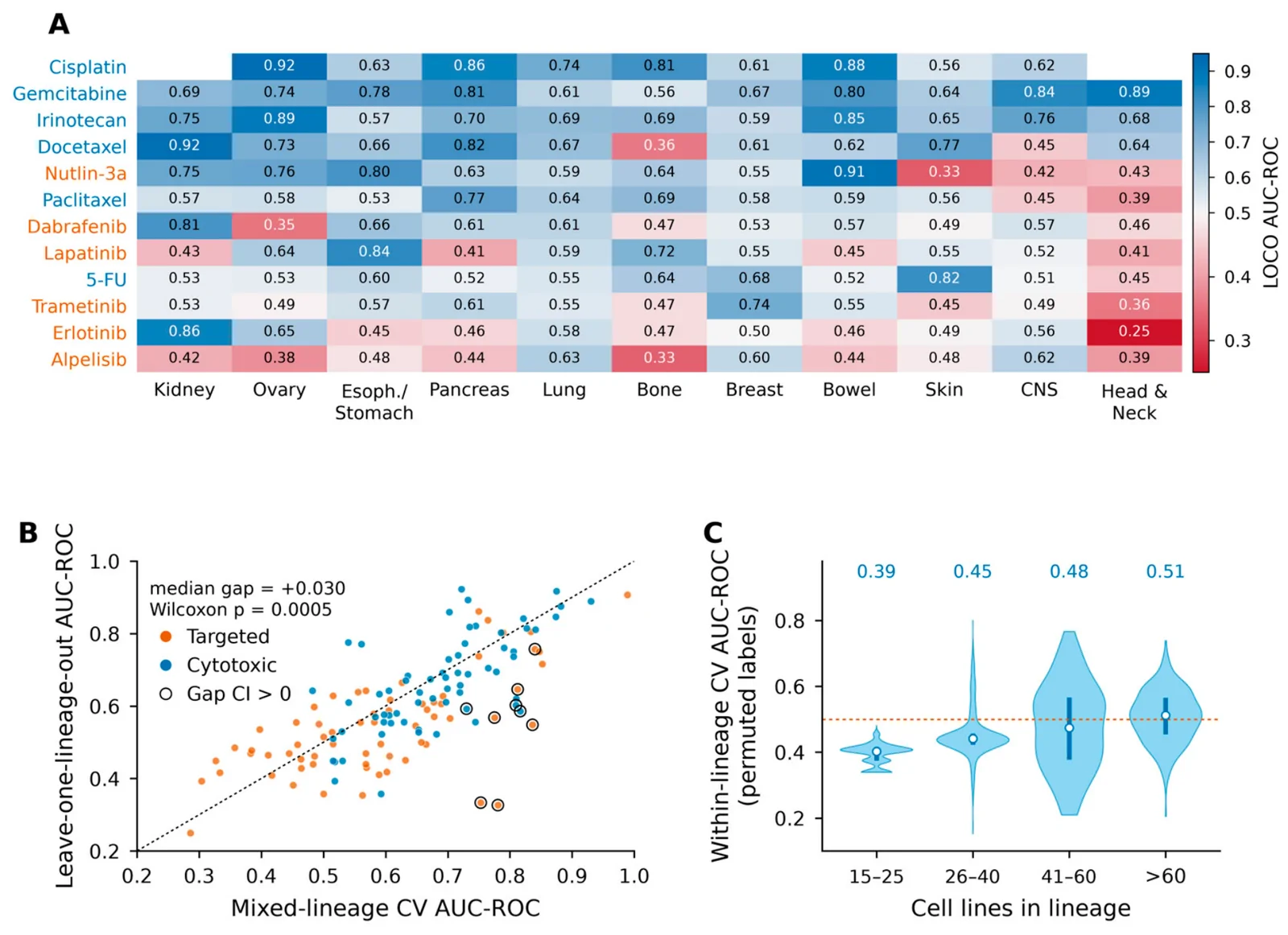
Cross-lineage transfer. (**A**) Leave-one-lineage-out (LOCO) AUC of the expression model for each drug and withheld solid lineage, using labels defined within each lineage; blank cells denote lineages with fewer than 15 cell lines for that drug. (**B**) LOCO AUC against mixed-lineage cross-validation AUC computed on the same cell lines for 130 drug-lineage pairs; circled points have a bootstrap 95% CI for the difference that excludes zero in favour of mixed-lineage cross-validation, and the dotted line is identity. (**C**) Distribution of within-lineage cross-validated AUC under permuted labels by lineage size (20 permutations per pair); numbers give the mean, the dashed line marks the nominal null value of 0.5, and bars show the interquartile range.

With labels defined within each lineage, LOCO AUCs for the expression model varied widely between drug-lineage pairs (Figure 4A). Median LOCO AUC across lineages was highest for cisplatin (0.740), gemcitabine (0.736) and irinotecan (0.691), and lowest for alpelisib (0.440) and erlotinib (0.495); by lineage it was highest for kidney (0.694) and lowest for head and neck (0.429), and 35 of 130 pairs fell below 0.5. These estimates were imprecise: the median width of the bootstrap 95% CI was 0.44 (0.50 for lineages with fewer than 25 cell lines and 0.28 for those with 40 or more). None of the 35 pairs below 0.5 had an interval entirely below 0.5, whereas 30 pairs had intervals entirely above 0.5 (Table S17). On the same cell lines, mixed-lineage cross-validation gave higher AUCs than LOCO in 85 of 130 pairs (median 0.642 versus 0.589; median paired difference +0.030, Wilcoxon *p* = 0.0005; Figure 4B). The paired bootstrap interval excluded zero in favour of mixed-lineage cross-validation for nine pairs and in favour of LOCO for four. Results for early fusion were similar (median difference +0.029, *p* = 0.004). Raising the minimum lineage size to 20 cell lines (eight lineages, 94 pairs) gave a median gap of +0.031 (*p* = 0.001) and to 25 cell lines (six lineages, 69 pairs) a gap of +0.027 (*p* = 0.011). With 30 or more cell lines only lung, breast and oesophagus and stomach remained (36 pairs), and the gap fell to +0.011 (*p* = 0.35). In the permutation control, within-lineage cross-validation with permuted labels gave a mean AUC of 0.431 (95% range 0.339 to 0.626) instead of the expected 0.5. The downward bias was largest in the smallest lineages (mean 0.394 for 15 to 25 cell lines and 0.507 for more than 60; Figure 4C), so within-lineage cross-validation is not a valid comparator at these sample sizes.

### 3.6. External Validation in an Independent Screen

**Figure 5:**
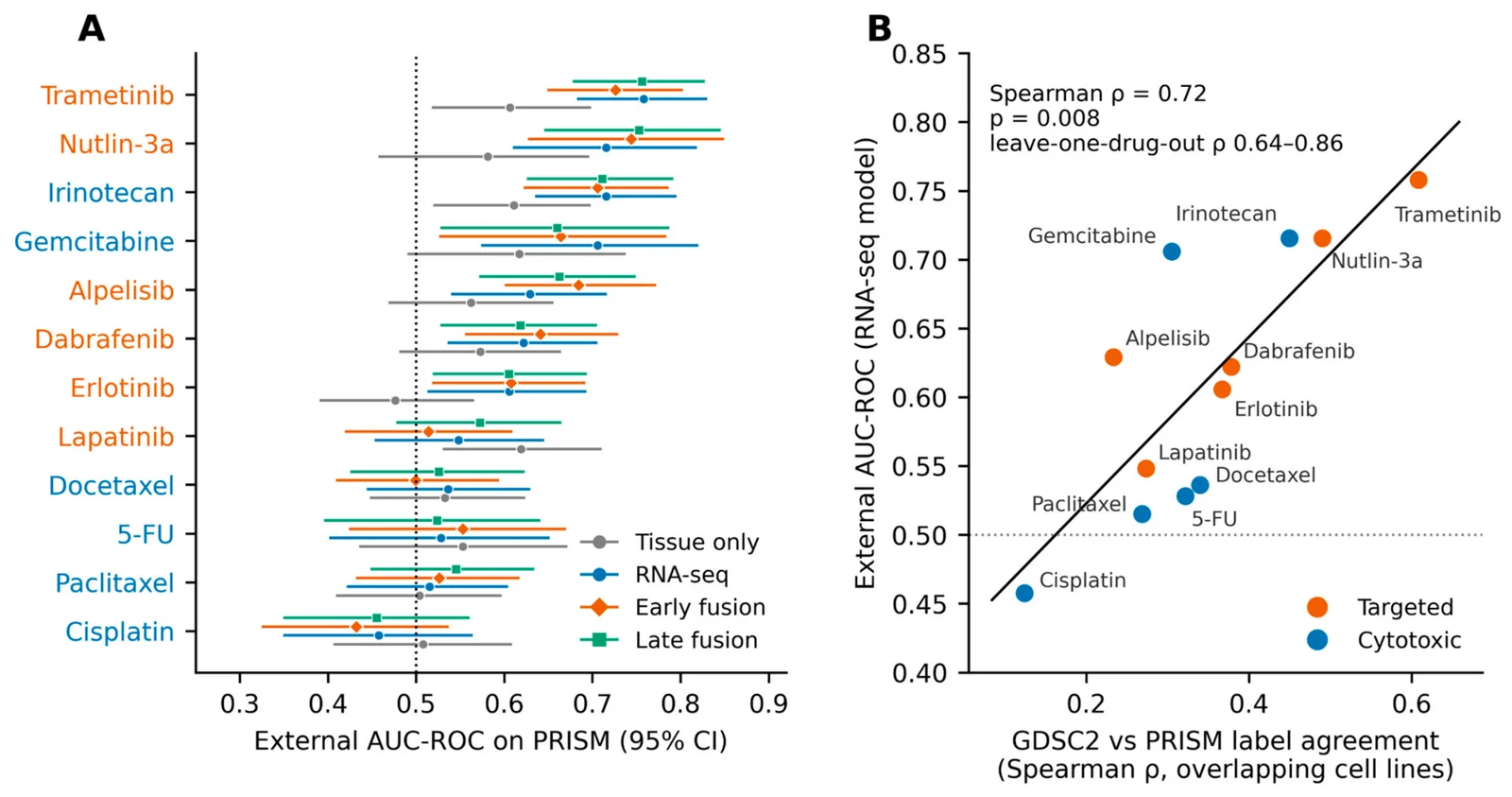
External validation on PRISM. (**A**) AUC with bootstrap 95% confidence intervals for the tissue-only, expression, early-fusion and late-fusion models trained on the GDSC2 and tested on unseen PRISM cell lines; the dotted line marks chance. Late-fusion values come from the external rebuild (Table S18); the other models show the published estimates (Table S10). (**B**) External expression-model AUC against the Spearman correlation between the GDSC2 and PRISM responses on cell lines measured in both screens; the line is a least-squares fit.

When models trained on the GDSC2 were applied to unseen PRISM cell lines, expression-model AUCs ranged from 0.458 (cisplatin) to 0.758 (trametinib), with a median of 0.614 (Figure 5A, Table S10). The highest external AUCs were for trametinib (0.758, 95% CI 0.684 to 0.829), nutlin-3a (0.716, 0.611 to 0.817), irinotecan (0.715, 0.637 to 0.794) and gemcitabine (0.706, 0.575 to 0.818), whereas the taxanes, 5-fluorouracil and cisplatin performed close to chance. Expression models exceeded the tissue-only model in nine of 12 drugs (median 0.614 versus 0.568), and early fusion exceeded expression in six of 12 (median 0.624), with the largest external fusion gain again for a targeted agent (alpelisib, 0.684 versus 0.629). Late fusion gave external AUCs of 0.455 to 0.756 (median 0.612) and exceeded the expression model of the same rebuild in eight of 12 drugs (Figure 5A, Table S18). In that rebuild, expression, early-fusion and tissue-only AUCs differed from the published values by a median of 0.008 (maximum 0.042), which indicates the seed-to-seed variability of single external estimates. Defining PRISM sensitivity with a threshold fixed outside the evaluation cohort changed the expression-model AUC by a median of 0.012 (overlap-based threshold) and 0.006 (PRISM-wide threshold), and the ordering of drugs was preserved (Spearman rho 0.98 and 0.99); the threshold-free correlation between predicted sensitivity and PRISM AUC ranged from 0.00 to 0.50. External AUC was strongly associated with agreement between the two screens on overlapping cell lines (Spearman rho = 0.72, *p* = 0.008; Figure 5B). The association held when any single drug was removed (rho 0.64 to 0.86, all *p* < 0.05) and after adjustment for internal cross-validated AUC (partial rho 0.63, *p* = 0.039), although the bootstrap CI over the 12 drugs was wide (0.14 to 1.00; Table S19): drugs whose GDSC2 and PRISM measurements agreed poorly, such as cisplatin (rho = 0.12), could not be predicted externally, whatever their internal performance.

### 3.7. Recovery of Established Pharmacogenomic Biomarkers

**Figure 6:**
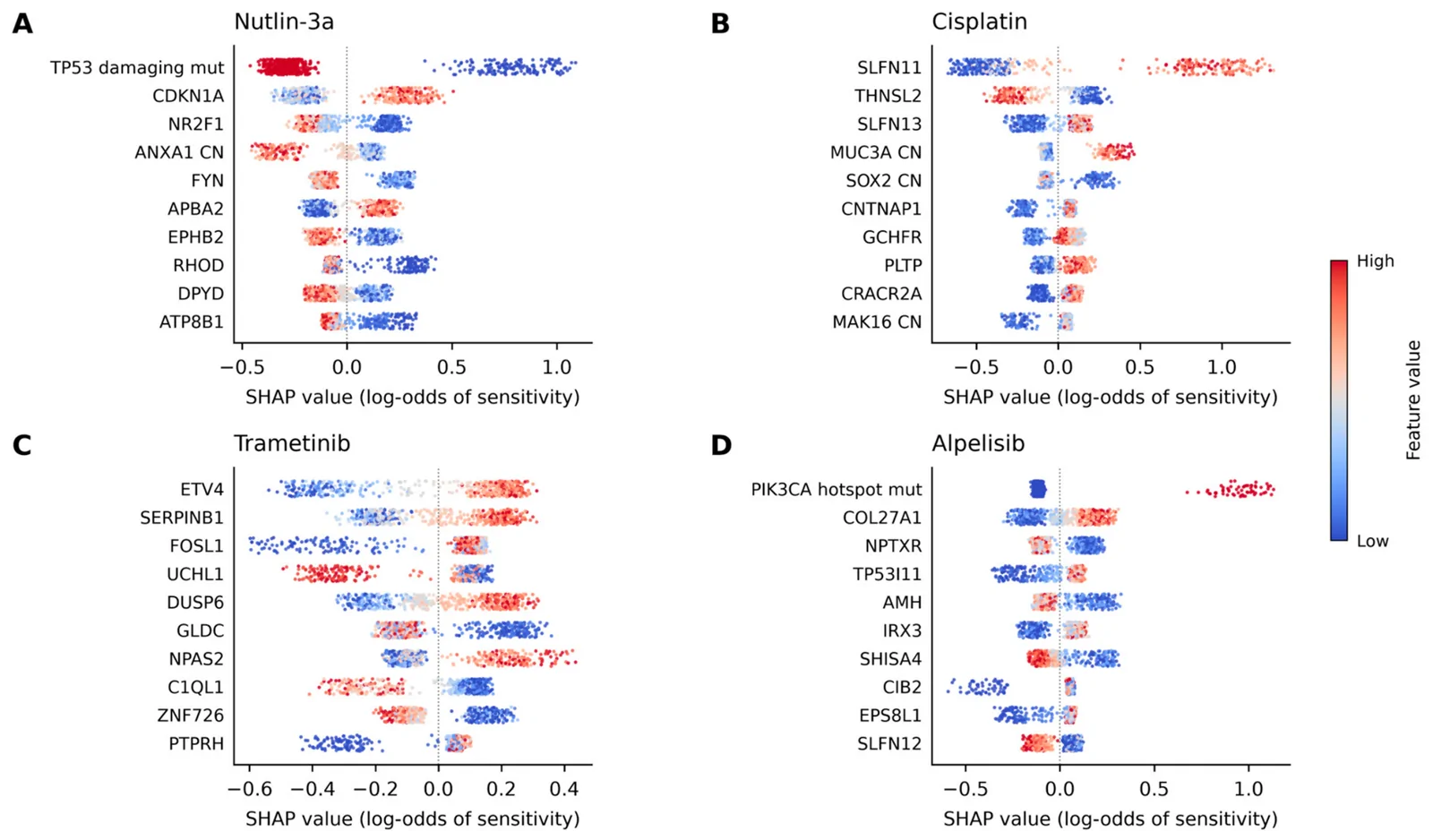
SHAP summaries for four representative drugs. (**A**) Nutlin-3a, (**B**) cisplatin, (**C**) trametinib and (**D**) alpelisib. The ten features with the largest mean absolute SHAP value in the early-fusion model are shown; each point is a cell line coloured by its feature value, and positive SHAP values indicate a higher predicted probability of sensitivity. Features without a suffix are expressions; CN, copy number; mut, mutation.

The highest-ranked features of the early-fusion models included several established determinants of drug response (Figure 6, Table S11). *SLFN11* expression was the top-ranked feature among more than 2300 for cisplatin, gemcitabine and irinotecan, with higher expression associated with sensitivity, and ranked 88th for 5-fluorouracil. For nutlin-3a, the *TP53* damaging mutation was the top feature and was associated with resistance, followed by expression of the p53 target *CDKN1A*, associated with sensitivity. The *BRAF* hotspot mutation ranked fourth for dabrafenib, and the *PIK3CA* hotspot mutation ranked first for alpelisib. For trametinib, the MAPK transcriptional outputs *ETV4*, *FOSL1* and *DUSP6* ranked first, third and fifth, whereas the *KRAS* and *BRAF* hotspot mutations contributed negligibly (ranks 1965 and 2331). The *EGFR* expression and copy number ranked 62nd and 91st for erlotinib, and the *ERBB2* copy number ranked 83rd for lapatinib. Two expected markers were not available to the models: the *ABCB1* expression for the taxanes and the *ERBB2* expression for lapatinib were not selected by the variance-based filter.

### 3.8. Sensitivity Analyses

Adding curated drug-specific biomarkers changed the AUC little (Table S16). For the expression model, the median change was +0.001 (range −0.014 to +0.025). The only significant gain with the DeLong test after the Holm correction was for paclitaxel (+0.025, adjusted *p* = 0.037), whose curated set includes the *ABCB1*, and it was not significant with the corrected resampled *t*-test (adjusted *p* = 0.55). For lapatinib, adding the *ERBB2* expression and the other curated genes changed the AUC by −0.002. For early fusion, the median change was +0.003, and no change survived correction. The dominance of expression therefore did not depend on the variance filter, although a single marker such as the *ABCB1* may matter for an individual drug. Alternative response definitions gave higher AUCs, as expected when intermediate cell lines are removed or the classes are more extreme (median expression-model AUC 0.799 for tertile extremes and 0.782 for the most sensitive quarter, against 0.739 for the median split; Table S15). Under both definitions, the expression model exceeded the tissue baseline for all 12 drugs (Holm-adjusted *p* < 0.05 for 11 and 12 drugs), early fusion again added little (median change +0.002 and −0.004; no drug significant after correction), and the ordering of drugs by expression-model AUC resembled that of the median split (Spearman rho 0.78 and 0.73). The OncoTree subtype baseline did not outperform the lineage baseline (median difference −0.042). With 58 categories, 31 of which contained fewer than five cell lines, the subtype indicators were sparse, and the finer labels lowered AUC for most drugs. Expression models exceeded the subtype baseline for every drug (median +0.137; Holm-adjusted *p* < 0.05 for all 12 with both tests), so the advantage of expression over tissue of origin is not explained by coarse lineage labels.

## 4. Discussion

Across the twelve therapeutic compounds examined in this work, baseline gene expression captured nearly all predictable drug sensitivity in cancer cell lines. Expression models consistently surpassed tissue-of-origin baselines across all agents, demonstrating statistically confirmed improvements for seven drugs. In contrast, incorporating somatic mutation and copy number alterations adjusted classification AUCs by no more than 0.035, with zero of 24 paired fusion evaluations retaining significance following family-wise error adjustment. Pooled across drugs, however, early fusion added a small average gain of 0.008 AUC that was not confirmed in a re-analysis, and the per-drug analyses could only detect gains of about 0.03 to 0.04 AUC, so our data indicate that any contributions from mutation and copy number alterations are small and largely drug-specific rather than broad. Discriminative accuracy dropped on unrepresented lineages and deteriorated further on an external screening platform, approaching chance levels for several cytotoxic agents. The magnitude of this cross-screen drop reflected the degree of experimental concordance between the underlying assays. These findings reflect a specific experimental scope: twelve compounds, three omics layers, two cell-line screening resources, and tree-based gradient boosting; caution is warranted before extrapolating them to all pharmacogenomic applications.

Our findings align with the early DREAM challenge benchmarks and subsequent pan-cancer investigations indicating that transcriptional variation explains vastly more pharmacological variance than static DNA alterations [5,9,10]. They likewise suggest caution regarding claims of sweeping multi-omics benefits from complex deep-learning architectures [11,12], whose evaluation schemes have frequently omitted paired statistical hypothesis testing or multiple-comparison adjustments across compounds. Still, genomic mutations remain informative in circumscribed biological contexts. For targeted therapies directed against specific oncogenic drivers, specifically nutlin-3a (*TP53*), dabrafenib (*BRAF*), and alpelisib (*PIK3CA*) mutation features garnered the highest SHAP attribution shares, generated the largest numerical late-fusion increments, and ranked prominently among key predictive markers. Mutation-only classifiers were numerically above the lineage baseline only for nutlin-3a and trametinib, and neither difference survived correction. This profile reflects the biological distinction between pathway-targeted therapies and broad cytotoxics; however, comparing six compounds per group yielded no statistically significant difference between drug classes, and verifying effects of this modest scale will require dramatically expanded screening panels.

Biologically, the predictive pre-eminence of RNA profiles suggests that the transcriptome operates as an integrative readout, reflecting cumulative consequences of multiple genomic lesions alongside epigenetic, metabolic, and post-transcriptional states that influence cellular drug sensitivity. Trametinib illustrates this principle clearly: the downstream MAPK transcriptional targets *ETV4*, *FOSL1*, and *DUSP6,* which constitute validated MEK-dependency signatures [31], occupied three of the top five feature ranks, whereas upstream *KRAS* and *BRAF* hotspot mutations contributed virtually no predictive weight. Pathway activation appears to be captured largely by transcriptional output. In a similar vein, the *SLFN11* expression, an established marker of susceptibility to DNA-damaging agents [32], emerged as the leading predictor for cisplatin, gemcitabine, and irinotecan, while the *TP53*-CDKN1A regulatory axis underlying MDM2 inhibition [33] was rediscovered for nutlin-3a. The mutation features already encode the mutated gene and its functional class, with separate indicators for hotspot or oncogenic high-impact variants and for damaging variants, and the models selected the expected ones: the *BRAF* hotspot indicator for dabrafenib, the *PIK3CA* hotspot indicator for alpelisib and the *TP53* damaging indicator for nutlin-3a (Table S11). Direct mutation indicators appeared to supply non-redundant signal primarily when an actionable lesion lacked a complete transcriptional surrogate, as seen with the *BRAF* V600 in dabrafenib response [34] or the *PIK3CA* mutations in alpelisib sensitivity [35]. The mechanism of action also helps explain why expression-model AUCs varied between drugs. Discrimination was highest for drugs whose response depends on programmes active across many cell lines, such as *SLFN11*-dependent responses to DNA damage for cisplatin, gemcitabine and irinotecan, MAPK pathway output for trametinib and p53 pathway integrity for nutlin-3a. It was lowest for dabrafenib, lapatinib, erlotinib and alpelisib, whose activity depends on a lesion present in a minority of cell lines (*BRAF* V600, *ERBB2* amplification, activating *EGFR* mutation, *PIK3CA* mutation). For these drugs, a median split labels many cell lines without the relevant lesion as sensitive, which limits what any molecular model can achieve.

Our cross-lineage experiments demonstrate that standard mixed-lineage cross-validation overestimates model transfer to unobserved tumour types. On strictly identical test sets, mixed cross-validation surpassed leave-one-lineage-out accuracy by a median optimism gap of 0.030 AUC. About a quarter of drug-lineage pairs had point estimates below 0.5, but none had a confidence interval entirely below 0.5, and individual estimates in lineages of 15 to 25 cell lines were too imprecise to interpret alone. Because labels were centred on lineage-specific medians, this optimism gap cannot be dismissed as simple tissue recognition; rather, it points to lineage-specific transcriptional contexts that algorithms learn only when that histology is represented during training. The gap was stable down to a minimum of 25 cell lines per lineage but smaller and not significant when only the three largest lineages were analysed; part of it may reflect small evaluation sets, and the estimate applies to solid tumours because haematological lineages were not evaluated. Lineage is also a coarse label that pools biologically distinct subtypes; replacing it with OncoTree subtypes did not change the conclusions (Section 3.8). Subtype-level transfer was not tested because only 10 to 12 subtypes per drug had 15 or more cell lines. Likewise, receptor-defined breast cancer subtypes could not be modelled separately: the 41 breast cancer cell lines comprised 21 TNBC, 16 HER2-positive and 4 HR-positive/HER2-negative lines, and some subtypes contained as few as zero to three sensitive lines per drug. These dynamics support the use of transfer learning and few-shot domain adaptation in preclinical pipeline design [13]. Our permutation benchmarks also uncovered a critical methodological artefact: standard stratified cross-validation within small lineages (15–25 cell lines) produced null AUC distributions shifted down to ~0.39, a recognised distortion of small-sample resampling [26]. Without an explicit permutation baseline, evaluating within-lineage splits would have created a misleading impression of cross-lineage transfer.

In independent validation, the strong association between inter-screen concordance and held-out PRISM accuracy indicates that much of the external performance attrition stems from assay discordance between screening repositories rather than outright model failure. The association rests on 12 drugs, and its bootstrap interval is wide, so it does not establish assay differences as the main cause; it was, however, stable when any one drug was removed and after adjustment for internal performance. Substantial disparities in screening duration, viability reagents, and concentration ranges between the GDSC2 and PRISM [15,16] plausibly limit cross-screen model portability. For drugs plagued by low inter-screen agreement, such as cisplatin and both taxanes, external accuracy hovered near chance regardless of internal cross-validation strength. By contrast, compounds exhibiting higher experimental concordance, including trametinib, nutlin-3a, and irinotecan, maintained external AUCs above 0.71 on unseen cell lines, which indicates that the learned rules capture biology reproducible across laboratories for these drugs. Prospective use would also require a sensitivity threshold fixed in advance; with thresholds set outside the evaluation cohort, the external results changed little (Section 3.6), but calibrated probabilities and drug-specific clinical cut-offs would be needed in practice.

From a practical perspective, these results suggest that for cell-line pharmacogenomic models of the kind studied here, deeper transcriptomic characterisation and lineage-diverse screening cohorts are likely to add more than indiscriminately layering further bulk genomic assays. Where driver mutations define how a drug acts, the corresponding mutation features should be retained; however, adding broad mutation and copy number panels by default yields diminishing returns. Preclinical oncology benchmarks should routinely report lineage-held-out transfer and cross-screen validation alongside conventional cross-validation, providing realistic appraisals of translational potential [14,17]. The findings rest on a uniform protocol applied to all twelve drugs, fold-internal preprocessing, paired testing with multiplicity control and explicit tissue-of-origin baselines.

Several biological boundaries must be noted. All models were developed and tested in immortalised two-dimensional cell lines cultured on plastic, which entirely lack the stromal, vascular, and immune architecture that profoundly dictates drug delivery and pharmacological efficacy in patient tumours, so the findings cannot be translated directly into claims of clinical efficacy. The responses modelled here are cell-autonomous: they measure how tumour cells respond to a drug in isolation. However, many clinically important effects depend on the tumour microenvironment. Immune checkpoint antibodies and other monoclonal antibodies act through immune effector mechanisms that are absent in culture [36], and tumour-associated macrophages, including through macrophage-derived long non-coding RNAs, shape immune evasion and treatment response [37]. Inflammatory signalling and microenvironmental mediators also influence tumour progression and drug activity [38]. None of these processes is represented in our features, no immunotherapy was tested, and the models should not be used to predict responses that depend on immune or stromal context. In addition, response was defined here by binary thresholds at the median LN_IC50. Regression analyses ranked the drugs almost identically (Spearman rho 0.92 between expression-model AUC and regression rho) and showed the same small fusion gains without significance after correction (Table S14); alternative thresholds did not change the main comparisons (Section 3.8), but binarisation inevitably simplifies the non-linear shape of drug sensitivity curves. Whether DNA methylation, chromatin accessibility or proteomic profiles add information beyond expression was not tested here. Proteomic and phosphoproteomic data could in principle capture pathway activity that RNA misses, but the value of any additional layer has to be measured directly because each layer can also add redundant or noisy features.

Some modelling and statistical choices also need comment. Hyperparameters were fixed in advance and not tuned per fold to prevent selection bias and optimistic performance estimates; this conservative choice may slightly lower peak achievable discrimination and need not affect all modalities equally. The unsupervised variance filter used to limit feature dimensionality also excluded known low-variance biomarkers, such as the *ABCB1* expression in taxane response and the *ERBB2* expression in lapatinib sensitivity; the *ERBB2* copy number was retained. Forcing curated biomarkers into the models improved AUC significantly only for paclitaxel and left the comparison between modalities unchanged (Section 3.8), so the filter did not drive the main conclusions, although curated priors may still help for individual drugs. The DeLong intervals are conditional on the training data; the corrected resampled *t*-test, which allows for overlapping training sets, was more conservative, confirmed the expression advantage over tissue for six drugs and found no fusion gain for any drug. Finally, leave-one-lineage-out estimates for smaller lineages (fewer than 25 cell lines) had confidence intervals around 0.5 AUC wide, so individual lineage-specific estimates should not be interpreted in isolation.

Looking ahead, translating preclinical response models toward clinical utility will require separating purely tumour-cell-intrinsic mechanisms from host and microenvironmental factors. While cell-autonomous models predict response from the baseline molecular state, clinical classifiers must incorporate systemic inflammatory signals, histopathology, imaging, and clinical parameter modalities increasingly integrated by multimodal AI pipelines in settings such as triple-negative breast cancer [39]. Another valuable direction involves employing multi-omics models to uncover sparse feature interactions [40], such as an activating driver mutation [41] contingent on a permissive transcriptional programme [42], that delineate drug sensitivity within specific tumour subtypes; this requires interaction-aware attribution and validation in independent cohorts. Finally, cell-line predictions such as those in Figure 4A are not directly comparable with standard-of-care practice or trial outcomes because the cell-line assay measures short-term viability in vitro, and several drug-lineage pairs are not used clinically; linking them would require patient-derived models or clinical cohorts with matched molecular data.

## 5. Conclusions

Across twelve cytotoxic and targeted therapies evaluated under strict multiplicity control, RNA expression profiles provided most of the predictive accuracy achievable from multi-omics data in cancer cell lines. Incorporating somatic mutations and copy number alterations yielded only marginal improvements that could not be confirmed for any individual drug after correction; pooled across drugs, early fusion added a small, unconfirmed average gain of around 0.01 AUC, and the largest numerical gains occurred for targeted agents whose targets carry specific oncogenic alterations. Conventional cross-validation substantially overestimated model portability across solid tumour lineages, and external accuracy tracked experimental concordance between screening assays. Preclinical pharmacogenomic modelling should therefore prioritise high-depth transcriptomic profiling and benchmark models rigorously against tissue baselines, unrepresented lineages, and independent screening platforms prior to claiming superior or transferable predictive performance. The conclusions apply to the drugs, cell-line resources and models examined and should be tested with larger drug panels and further molecular layers.

## Figures and Tables

**Table 1:** Study drugs, cohort sizes and agreement between screens.

Drug	Target or Mechanism	Class	GDSC2 Cell Lines (*n*)	Median LN_IC50	PRISM External Lines (*n*)	GDSC2 vs. PRISM Rho (Overlap *n*)
Docetaxel	Microtubule stabiliser	Cytotoxic	509	−3.74	158	0.34 (367)
Paclitaxel	Microtubule stabiliser	Cytotoxic	505	−3.31	159	0.27 (367)
Cisplatin	DNA crosslinking	Cytotoxic	429	3.20	119	0.12 (231)
Gemcitabine	Nucleoside analogue	Cytotoxic	507	−0.99	77	0.30 (221)
5-Fluorouracil	Thymidylate synthase	Cytotoxic	509	4.53	89	0.32 (246)
Irinotecan	Topoisomerase I	Cytotoxic	508	2.52	162	0.45 (363)
Nutlin-3a	MDM2	Targeted	509	4.94	89	0.49 (255)
Dabrafenib	BRAF V600	Targeted	502	4.66	153	0.38 (334)
Trametinib	MEK1/2	Targeted	508	0.41	163	0.61 (374)
Lapatinib	EGFR/ERBB2	Targeted	508	3.13	152	0.27 (361)
Erlotinib	EGFR	Targeted	502	2.89	163	0.37 (367)
Alpelisib	PI3K alpha	Targeted	508	3.46	157	0.23 (358)

LN_IC50, natural logarithm of the half-maximal inhibitory concentration (micromolar). PRISM external lines are cell lines with DepMap omics data that were not part of the GDSC2 training set for that drug. Rho is the Spearman correlation between the GDSC2 LN_IC50 and PRISM area under the dose–response curve on cell lines measured in both screens.

## Data Availability

All data analysed in this study are publicly available from the DepMap portal (release 26Q1, https://depmap.org/portal; accessed on 22 May 2026), the Cell Model Passports resource (GDSC2 fitted dose–response, release 27 October 2023, https://cellmodelpassports.sanger.ac.uk) and the PRISM Repurposing secondary screen (release 20Q2, https://depmap.org/repurposing accessed on 22 May 2026). Analysis code with fixed random seeds and data-access and run instructions is available at https://github.com/Babajan-B/Multi-Omics-CDSR. The drug-cell line inclusion table, the features selected for each drug and the curated biomarker lists are provided in the Supplementary Materials (Tables S20–S22).

## Supplementary Materials

The following supporting information can be downloaded at: https://www.mdpi.com/article/10.3390/cancers18213503/s1. Table S1: Internal cross-validated AUC with 95% CI and repeat standard deviation for all seven models and twelve drugs; Table S2: All paired DeLong comparisons with raw and Holm-adjusted *p* values; Table S3: Leave-one-lineage-out and mixed-lineage cross-validation AUCs for all 260 drug-lineage-model combinations; Table S4: Permutation control results (2600 runs); Table S5: External validation results; Table S6: Regression performance; Table S7: Ranks of pre-specified biomarkers; Table S8: Internal cross-validated performance by drug; Table S9: Paired comparisons of expression models with tissue-only and fusion models; Table S10: External validation on PRISM; Table S11: Ranks of biomarkers in early-fusion SHAP attribution; Table S12: Pooled fusion gains, equivalence bounds and minimum detectable differences; Table S13: Per-repeat DeLong tests and corrected resampled *t*-tests; Table S14: Paired tests of regression fusion gains; Table S15: Alternative response definitions; Table S16: Curated biomarkers and OncoTree subtype baseline; Table S17a: Leave-one-lineage-out AUC of the expression model with bootstrap 95% CI for every drug-lineage pair; Table S17b: LOCO uncertainty summarised by withheld lineage (expression model); Table S17c: Effect of the minimum lineage size on the mixed-lineage versus LOCO comparison. Table S18: Rebuilt external validation with late fusion and alternative PRISM thresholds; Table S19: Robustness of the concordance association; Table S20: Curated biomarker lists; Table S21: Drug-cell line inclusion table; Table S22: Features selected for each drug.

## Abbreviations

AUC, area under the receiver operating characteristic curve; CI, confidence interval; CN, copy number; DepMap, Cancer Dependency Map; GDSC, Genomics of Drug Sensitivity in Cancer; LN_IC50, natural logarithm of the half-maximal inhibitory concentration; LOCO, leave-one-lineage-out; MUT, mutation; SHAP, SHapley Additive exPlanations; TPM, transcripts per million.
