# Supplementary material for: Transcriptomic Profiles Carry Most of the Multi-Omics Signal for Drug Sensitivity Prediction in Cancer Cell Lines: A Cross-Lineage and Independent-Screen Evaluation of Twelve Anticancer Agents
Source: Cancers (Basel). 2026 Oct 30;18(21):3503. doi: 10.3390/cancers18213503 (PMC13761415; doi:10.3390/cancers18213503)
Supplement: Supplementary file 1 [file cancers-18-03503-s001.zip › Supplementary_Tables_S8-S20.pdf]

Supplementary Tables S8 to S22

Transcriptomic Profiles Carry Most of the Multi-Omics Signal for Drug Sensitivity Prediction in Cancer Cell Lines. Tables S1 to S7 are provided as sheets in the accompanying Excel workbook, which also contains every table below.

Table S8. Internal cross-validated performance by drug.

Drug	Tissue only AUC (95% CI)	RNA AUC (95% CI)	Early fusion AUC (95% CI)	Late fusion AUC (95% CI)	RNA regression rho (95% CI)
Docetaxel	0.640 (0.592-0.687)	0.740 (0.698-0.783)	0.725 (0.682-0.769)	0.733 (0.690-0.776)	0.46 (0.38-0.53)
Paclitaxel	0.607 (0.557-0.656)	0.714 (0.670-0.758)	0.699 (0.654-0.744)	0.707 (0.663-0.752)	0.45 (0.38-0.53)
Cisplatin	0.665 (0.614-0.716)	0.777 (0.733-0.821)	0.788 (0.745-0.830)	0.792 (0.751-0.834)	0.61 (0.53-0.67)
Gemcitabine	0.624 (0.575-0.672)	0.793 (0.755-0.832)	0.816 (0.780-0.852)	0.795 (0.757-0.833)	0.61 (0.54-0.67)
5-Fluorouracil	0.675 (0.629-0.722)	0.730 (0.687-0.774)	0.749 (0.707-0.792)	0.734 (0.691-0.777)	0.47 (0.40-0.54)
Irinotecan	0.654 (0.607-0.702)	0.810 (0.773-0.847)	0.815 (0.779-0.852)	0.807 (0.770-0.844)	0.66 (0.60-0.72)
Nutlin-3a	0.677 (0.631-0.724)	0.809 (0.772-0.846)	0.825 (0.790-0.861)	0.830 (0.795-0.865)	0.66 (0.60-0.71)
Dabrafenib	0.667 (0.620-0.714)	0.699 (0.653-0.745)	0.735 (0.691-0.778)	0.725 (0.680-0.769)	0.50 (0.42-0.57)
Trametinib	0.703 (0.658-0.749)	0.836 (0.802-0.870)	0.837 (0.803-0.871)	0.837 (0.803-0.872)	0.67 (0.62-0.72)
Lapatinib	0.666 (0.619-0.713)	0.690 (0.644-0.736)	0.693 (0.648-0.739)	0.691 (0.645-0.737)	0.42 (0.35-0.50)
Erlotinib	0.631 (0.582-0.679)	0.692 (0.646-0.739)	0.707 (0.662-0.753)	0.697 (0.651-0.743)	0.39 (0.31-0.48)
Alpelisib	0.638 (0.590-0.686)	0.698 (0.653-0.744)	0.716 (0.671-0.760)	0.718 (0.674-0.763)	0.39 (0.31-0.46)

AUC, area under the receiver operating characteristic curve with DeLong 95% CI. Regression rho is the Spearman correlation between predicted and observed negative LN_IC50 with a bootstrap 95% CI. All models used XGBoost.

Table S9. Paired comparisons of expression models with tissue-only and fusion models.

Drug	RNA minus tissue (delta AUC)	Holm p	Early minus RNA (95% CI)	p	Holm p	Late minus RNA (95% CI)	p	Holm p
Docetaxel	+0.100	<0.0001	-0.015 (-0.032 to +0.002)	0.079	1.000	-0.008 (-0.018 to +0.003)	0.161	1.000
Paclitaxel	+0.107	0.0003	-0.015 (-0.031 to +0.002)	0.079	1.000	-0.006 (-0.013 to 0.000)	0.061	1.000
Cisplatin	+0.112	<0.0001	+0.011 (-0.006 to +0.028)	0.194	1.000	+0.016 (+0.001 to +0.030)	0.038	0.803
Gemcitabine	+0.169	<0.0001	+0.023 (+0.005 to +0.041)	0.015	0.338	+0.002 (-0.009 to +0.013)	0.786	1.000
5-Fluorouracil	+0.055	0.063	+0.019 (-0.004 to +0.042)	0.098	1.000	+0.004 (-0.013 to +0.021)	0.652	1.000
Irinotecan	+0.156	<0.0001	+0.005 (-0.008 to +0.018)	0.426	1.000	-0.003 (-0.013 to +0.006)	0.526	1.000

Nutlin-3a	+0.132	<0.0001	+0.016 (-0.002 to +0.035)	0.086	1.000	+0.021 (+0.001 to +0.040)	0.040	0.803
Dabrafenib	+0.032	0.323	+0.035 (+0.012 to +0.059)	0.003	0.081	+0.025 (+0.003 to +0.048)	0.028	0.625
Trametinib	+0.133	<0.0001	+0.001 (-0.011 to +0.012)	0.884	1.000	+0.001 (-0.009 to +0.012)	0.792	1.000
Lapatinib	+0.024	0.340	+0.003 (-0.018 to +0.025)	0.749	1.000	+0.001 (-0.014 to +0.015)	0.923	1.000
Erlotinib	+0.062	0.063	+0.015 (-0.007 to +0.036)	0.174	1.000	+0.004 (-0.011 to +0.020)	0.587	1.000
Alpelisib	+0.060	0.054	+0.017 (-0.004 to +0.039)	0.114	1.000	+0.020 (-0.003 to +0.042)	0.087	1.000

Delta AUC values are paired differences on identical cell lines. p values are from the two-sided DeLong test; Holm p values are adjusted across the twelve drugs within each comparison family.

Table S10. External validation on PRISM cell lines not used for training.

**Drug**	**External lines (n)**	**Tissue only AUC (95% CI)**	**RNA AUC (95% CI)**	**Early fusion AUC (95% CI)**	**RNA prediction vs PRISM AUC (rho)**
Docetaxel	158	0.533 (0.449-0.622)	0.536 (0.445-0.628)	0.499 (0.411-0.593)	0.08
Paclitaxel	159	0.504 (0.411-0.596)	0.515 (0.423-0.603)	0.526 (0.433-0.616)	0.02
Cisplatin	119	0.508 (0.408-0.607)	0.458 (0.351-0.563)	0.432 (0.326-0.536)	-0.04
Gemcitabine	77	0.617 (0.492-0.736)	0.706 (0.575-0.818)	0.664 (0.528-0.783)	0.37
5-Fluorouracil	89	0.553 (0.437-0.670)	0.528 (0.403-0.650)	0.553 (0.425-0.669)	0.10
Irinotecan	162	0.611 (0.521-0.697)	0.715 (0.637-0.794)	0.706 (0.624-0.785)	0.44
Nutlin-3a	89	0.581 (0.459-0.695)	0.716 (0.611-0.817)	0.744 (0.628-0.848)	0.43
Dabrafenib	153	0.573 (0.483-0.663)	0.622 (0.537-0.704)	0.641 (0.557-0.728)	0.32
Trametinib	163	0.607 (0.519-0.697)	0.758 (0.684-0.829)	0.726 (0.650-0.801)	0.52
Lapatinib	152	0.619 (0.532-0.709)	0.548 (0.454-0.644)	0.514 (0.421-0.608)	0.15
Erlotinib	163	0.476 (0.392-0.564)	0.606 (0.515-0.692)	0.608 (0.520-0.690)	0.20
Alpelisib	157	0.562 (0.470-0.654)	0.629 (0.541-0.715)	0.684 (0.602-0.771)	0.34

AUC confidence intervals are from 2000 bootstrap resamples. The final column is the Spearman correlation between the predicted probability of sensitivity and the negative PRISM area under the dose-response curve.

Table S11. Ranks of pre-specified biomarkers in early-fusion SHAP attribution.

**Drug**	**Feature**	**Data type**	**Rank (of all features)**	**Direction of effect**
Cisplatin	SLFN11	Expression	1 of 2471	Sensitivity
Gemcitabine	SLFN11	Expression	1 of 2361	Sensitivity
Irinotecan	SLFN11	Expression	1 of 2361	Sensitivity
5-Fluorouracil	SLFN11	Expression	88 of 2363	Sensitivity
Nutlin-3a	TP53 damaging mutation	Mutation	1 of 2363	Resistance

Nutlin-3a	CDKN1A	Expression	2 of 2363	Sensitivity
Nutlin-3a	TP53 hotspot mutation	Mutation	17 of 2363	Resistance
Dabrafenib	BRAF hotspot mutation	Mutation	4 of 2359	Sensitivity
Trametinib	ETV4	Expression	1 of 2361	Sensitivity
Trametinib	FOSL1	Expression	3 of 2361	Sensitivity
Trametinib	DUSP6	Expression	5 of 2361	Sensitivity
Trametinib	KRAS hotspot mutation	Mutation	1965 of 2361	Negligible
Alpelisib	PIK3CA hotspot mutation	Mutation	1 of 2361	Sensitivity
Erlotinib	EGFR	Expression	62 of 2351	Sensitivity
Erlotinib	EGFR	Copy number	91 of 2351	Sensitivity
Lapatinib	ERBB2	Copy number	83 of 2361	Sensitivity
Lapatinib	ERBB2	Expression	not selected (of 2361)	Not applicable
Docetaxel	ABCB1	Expression	not selected (of 2363)	Not applicable

Direction is sensitivity when higher feature values (or presence of the mutation) increase the predicted probability of sensitivity. Not selected indicates that the gene did not pass the training-set variance filter. Supporting evidence: SLFN11 [32]; TP53 and CDKN1A [33]; BRAF [34]; ETV4, FOSL1 and DUSP6 [31]; PIK3CA [35].

Table S12. Fusion gains relative to the expression model: per-drug confidence intervals, equivalence bounds and minimum detectable differences.

Drug	Comparison	Delta AUC	95% CI	90% CI	90% CI within ±0.03	MDD80 (alpha 0.05)	MDD80 (alpha 0.05/12)
Docetaxel	Early minus RNA	-0.015	-0.032 to +0.002	-0.029 to -0.001	yes	0.024	0.032
Paclitaxel	Early minus RNA	-0.015	-0.031 to +0.002	-0.029 to -0.001	yes	0.024	0.031
Cisplatin	Early minus RNA	+0.011	-0.006 to +0.028	-0.003 to +0.025	yes	0.024	0.032
Gemcitabine	Early minus RNA	+0.023	+0.005 to +0.041	+0.007 to +0.039	no	0.026	0.035
5-Fluorouracil	Early minus RNA	+0.019	-0.004 to +0.042	+0.000 to +0.038	no	0.032	0.043
Irinotecan	Early minus RNA	+0.005	-0.008 to +0.018	-0.006 to +0.016	yes	0.018	0.024
Nutlin-3a	Early minus RNA	+0.016	-0.002 to +0.035	+0.001 to +0.032	no	0.026	0.035
Dabrafenib	Early minus RNA	+0.035	+0.012 to +0.059	+0.016 to +0.055	no	0.034	0.045
Trametinib	Early minus RNA	+0.001	-0.011 to +0.012	-0.009 to +0.010	yes	0.016	0.022
Lapatinib	Early minus RNA	+0.003	-0.018 to +0.025	-0.014 to +0.021	yes	0.030	0.040

Erlotinib	Early minus RNA	+0.015	-0.007 to +0.036	-0.003 to +0.033	no	0.031	0.040
Alpelisib	Early minus RNA	+0.017	-0.004 to +0.039	-0.001 to +0.036	no	0.031	0.041
Docetaxel	Late minus RNA	-0.008	-0.018 to +0.003	-0.017 to +0.001	yes	0.015	0.020
Paclitaxel	Late minus RNA	-0.006	-0.013 to +0.000	-0.012 to -0.001	yes	0.009	0.012
Cisplatin	Late minus RNA	+0.016	+0.001 to +0.030	+0.003 to +0.028	yes	0.021	0.028
Gemcitabine	Late minus RNA	+0.002	-0.009 to +0.013	-0.008 to +0.011	yes	0.016	0.021
5-Fluorouracil	Late minus RNA	+0.004	-0.013 to +0.021	-0.010 to +0.018	yes	0.024	0.032
Irinotecan	Late minus RNA	-0.003	-0.013 to +0.006	-0.011 to +0.005	yes	0.014	0.018
Nutlin-3a	Late minus RNA	+0.021	+0.001 to +0.040	+0.004 to +0.037	no	0.028	0.037
Dabrafenib	Late minus RNA	+0.025	+0.003 to +0.048	+0.006 to +0.044	no	0.032	0.043
Trametinib	Late minus RNA	+0.001	-0.009 to +0.012	-0.008 to +0.011	yes	0.016	0.021
Lapatinib	Late minus RNA	+0.001	-0.014 to +0.015	-0.011 to +0.013	yes	0.020	0.027
Erlotinib	Late minus RNA	+0.004	-0.011 to +0.020	-0.009 to +0.018	yes	0.023	0.030
Alpelisib	Late minus RNA	+0.020	-0.003 to +0.042	+0.001 to +0.039	no	0.032	0.043

Delta AUC and standard errors are from the paired DeLong test on out-of-fold predictions averaged over three repeats. MDD80, smallest difference detectable with 80% power at the stated two-sided alpha (0.05/12 is the strictest Holm threshold). Random-effects pooled difference (DerSimonian-Laird): early fusion +0.008 (95% CI +0.000 to +0.016, p=0.041, I2=59%); late fusion +0.003 (95% CI -0.002 to +0.009, p=0.26, I2=52%). Wilcoxon signed-rank test across the 12 drugs: early fusion p=0.034, late fusion p=0.129.

Table S13. Inference that accounts for the resampling design: DeLong comparisons within each original repeat, and the Nadeau-Bengio corrected resampled t-test on fold-level AUC differences from a re-run with new random partitions.

Drug	Comparison	Original: delta, range over 3 repeats	Original: DeLong p, range over repeats	Re-run: delta (averaged OOF)	Re-run: DeLong p	Re-run: DeLong Holm p	Re-run: mean fold delta (95% CI)	Re-run: corrected t p	Re-run: corrected t Holm p
Docetaxel	Early minus RNA	-0.021 to -0.005	0.037 to 0.653	-0.014	0.094	0.754	-0.011 (-0.036 to +0.014)	0.367	1.000

Paclitaxel	Early minus RNA	-0.027 to -0.009	0.011 to 0.401	-0.023	0.007	0.089	-0.025 (-0.052 to +0.003)	0.074	0.886
Cisplatin	Early minus RNA	+0.001 to +0.018	0.078 to 0.908	+0.006	0.475	1.000	+0.006 (-0.016 to +0.027)	0.587	1.000
Gemcitabine	Early minus RNA	+0.016 to +0.029	0.008 to 0.141	+0.018	0.061	0.547	+0.016 (-0.009 to +0.041)	0.187	1.000
5-Fluorouracil	Early minus RNA	+0.011 to +0.024	0.055 to 0.396	+0.017	0.144	1.000	+0.016 (-0.016 to +0.049)	0.307	1.000
Irinotecan	Early minus RNA	+0.001 to +0.008	0.325 to 0.901	-0.001	0.903	1.000	-0.001 (-0.020 to +0.018)	0.913	1.000
Nutlin-3a	Early minus RNA	+0.014 to +0.024	0.030 to 0.193	+0.018	0.039	0.430	+0.019 (-0.007 to +0.044)	0.136	1.000
Dabrafenib	Early minus RNA	+0.017 to +0.046	<0.001 to 0.219	+0.010	0.434	1.000	+0.009 (-0.017 to +0.035)	0.458	1.000
Trametinib	Early minus RNA	-0.004 to +0.004	0.551 to 0.885	-0.003	0.652	1.000	-0.002 (-0.019 to +0.015)	0.777	1.000
Lapatinib	Early minus RNA	-0.010 to +0.012	0.369 to 0.882	+0.011	0.319	1.000	+0.007 (-0.024 to +0.038)	0.630	1.000
Erlotinib	Early minus RNA	-0.005 to +0.021	0.110 to 0.691	+0.021	0.043	0.432	+0.015 (-0.013 to +0.043)	0.276	1.000
Alpelisib	Early minus RNA	+0.012 to +0.023	0.076 to 0.371	+0.007	0.552	1.000	+0.006 (-0.023 to +0.034)	0.676	1.000
Docetaxel	Late minus RNA	-0.012 to -0.007	0.050 to 0.315	-0.007	0.148	1.000	-0.006 (-0.033 to +0.022)	0.677	1.000
Paclitaxel	Late minus RNA	-0.012 to -0.005	0.015 to 0.276	-0.007	0.036	0.392	-0.008 (-0.023 to +0.008)	0.324	1.000
Cisplatin	Late minus RNA	+0.006 to +0.018	0.021 to 0.524	-0.000	0.990	1.000	+0.000 (-0.029 to +0.029)	0.996	1.000
Gemcitabine	Late minus RNA	-0.002 to +0.000	0.693 to 0.988	+0.001	0.833	1.000	-0.000 (-0.018 to +0.017)	0.985	1.000

5-Fluorouracil	Late minus RNA	-0.010 to +0.006	0.354 to 0.708	+0.006	0.482	1.000	+0.010 (-0.014 to +0.035)	0.379	1.000
Irinotecan	Late minus RNA	-0.012 to -0.001	0.030 to 0.928	-0.004	0.335	1.000	-0.001 (-0.012 to +0.011)	0.908	1.000
Nutlin-3a	Late minus RNA	+0.019 to +0.030	0.004 to 0.071	+0.022	0.023	0.274	+0.024 (-0.009 to +0.058)	0.143	1.000
Dabrafenib	Late minus RNA	+0.022 to +0.023	0.047 to 0.084	+0.013	0.273	1.000	+0.017 (-0.007 to +0.042)	0.151	1.000
Trametinib	Late minus RNA	-0.003 to +0.003	0.642 to 0.736	-0.001	0.864	1.000	+0.001 (-0.016 to +0.017)	0.925	1.000
Lapatinib	Late minus RNA	-0.011 to +0.005	0.212 to 0.958	+0.001	0.868	1.000	-0.001 (-0.020 to +0.018)	0.902	1.000
Erlotinib	Late minus RNA	-0.004 to +0.010	0.255 to 0.872	+0.002	0.720	1.000	-0.001 (-0.023 to +0.022)	0.961	1.000
Alpelisib	Late minus RNA	+0.011 to +0.029	0.029 to 0.378	+0.016	0.176	1.000	+0.014 (-0.018 to +0.046)	0.360	1.000
Docetaxel	RNA minus Tissue	+0.077 to +0.099	<0.001 to 0.001	+0.106	<0.001	<0.001	+0.096 (+0.054 to +0.138)	<0.001	0.002
Paclitaxel	RNA minus Tissue	+0.086 to +0.119	<0.001 to 0.002	+0.106	<0.001	<0.001	+0.087 (+0.005 to +0.168)	0.038	0.192
Cisplatin	RNA minus Tissue	+0.092 to +0.112	<0.001 to <0.001	+0.124	<0.001	<0.001	+0.107 (+0.044 to +0.170)	0.003	0.021
Gemcitabine	RNA minus Tissue	+0.149 to +0.170	<0.001 to <0.001	+0.176	<0.001	<0.001	+0.157 (+0.096 to +0.218)	<0.001	<0.001
5-Fluorouracil	RNA minus Tissue	+0.042 to +0.048	0.039 to 0.068	+0.072	0.002	0.007	+0.053 (-0.001 to +0.108)	0.053	0.211
Irinotecan	RNA minus Tissue	+0.143 to +0.148	<0.001 to <0.001	+0.171	<0.001	<0.001	+0.155 (+0.106 to +0.203)	<0.001	<0.001
Nutlin-3a	RNA minus Tissue	+0.111 to +0.129	<0.001 to <0.001	+0.138	<0.001	<0.001	+0.121 (+0.079 to +0.163)	<0.001	<0.001

Dabrafenib	RNA minus Tissue	+0.019 to +0.034	0.126 to 0.432	+0.044	0.060	0.120	+0.020 (-0.026 to +0.065)	0.376	0.751
Trametinib	RNA minus Tissue	+0.117 to +0.119	<0.001 to <0.001	+0.122	<0.001	<0.001	+0.109 (+0.045 to +0.173)	0.003	0.021
Lapatinib	RNA minus Tissue	+0.003 to +0.027	0.305 to 0.912	+0.042	0.114	0.120	+0.024 (-0.045 to +0.093)	0.470	0.751
Erlotinib	RNA minus Tissue	+0.035 to +0.071	0.010 to 0.203	+0.084	0.001	0.005	+0.073 (+0.016 to +0.129)	0.015	0.090
Alpelisib	RNA minus Tissue	+0.037 to +0.053	0.038 to 0.128	+0.056	0.015	0.046	+0.035 (-0.020 to +0.090)	0.190	0.570

Original: the three repeats of the published analysis, each tested separately with the paired DeLong test. Re-run: three repeats of stratified five-fold cross-validation with new random seeds; the corrected resampled t-test uses the 15 fold-level paired AUC differences with variance inflated by n_test/n_train. Holm adjustment is across the 12 drugs within each comparison.

Table S14. Regression (continuous negative LN_IC50) performance of expression and early-fusion models and paired tests of the change.

**Drug**	**Class**	**Spearman rho, RNA**	**Spearman rho, early fusion**	**Change**	**95% CI (paired bootstrap)**	**p**	**Holm p**
Docetaxel	Cytotoxic	0.460	0.492	+0.032	-0.011 to +0.074	0.144	1.000
Paclitaxel	Cytotoxic	0.454	0.429	-0.025	-0.067 to +0.016	0.222	1.000
Cisplatin	Cytotoxic	0.606	0.606	+0.000	-0.037 to +0.038	0.974	1.000
Gemcitabine	Cytotoxic	0.610	0.621	+0.011	-0.024 to +0.045	0.551	1.000
5-Fluorouracil	Cytotoxic	0.466	0.488	+0.022	-0.019 to +0.064	0.312	1.000
Irinotecan	Cytotoxic	0.663	0.646	-0.016	-0.040 to +0.005	0.143	1.000
Nutlin-3a	Targeted	0.660	0.679	+0.019	-0.016 to +0.054	0.300	1.000
Dabrafenib	Targeted	0.502	0.560	+0.058	+0.013 to +0.106	0.013	0.156
Trametinib	Targeted	0.668	0.677	+0.009	-0.015 to +0.035	0.477	1.000
Lapatinib	Targeted	0.423	0.453	+0.030	-0.012 to +0.072	0.171	1.000

Erlotinib	Targeted	0.394	0.429	+0.035	-0.011 to +0.081	0.148	1.000
Alpelisib	Targeted	0.386	0.432	+0.046	+0.001 to +0.093	0.049	0.539

2000 paired bootstrap resamples of cell lines. Early fusion increased rho in 10 of 12 drugs (median change +0.020; Wilcoxon signed-rank p=0.027); 2 changes were nominally significant and none after Holm correction.

Table S15. Internal AUC under alternative response definitions (re-run cross-validation).

Drug	Median Tissue	Median RNA	Median Early	Tertile Tissue	Tertile RNA	Tertile Early	Quartile Tissue	Quartile RNA	Quartile Early	Tertile RNA-Tissue (Holm p)	Tertile Early-RNA (Holm p)	Quartile RNA-Tissue (Holm p)	Quartile Early-RNA (Holm p)
Docetaxel	0.632	0.738	0.724	0.640	0.793	0.794	0.585	0.730	0.709	+0.153 (<0.001)	+0.001 (1.000)	+0.145 (<0.001)	-0.021 (0.710)
Paclitaxel	0.610	0.716	0.694	0.622	0.788	0.784	0.619	0.757	0.747	+0.167 (<0.001)	-0.004 (1.000)	+0.138 (<0.001)	-0.010 (1.000)
Cisplatin	0.667	0.792	0.797	0.680	0.888	0.894	0.696	0.865	0.861	+0.208 (<0.001)	+0.007 (1.000)	+0.169 (<0.001)	-0.004 (1.000)
Gemcitabine	0.618	0.794	0.813	0.676	0.872	0.885	0.684	0.806	0.802	+0.196 (<0.001)	+0.013 (0.847)	+0.123 (<0.001)	-0.004 (1.000)
5-Fluorouracil	0.669	0.740	0.757	0.663	0.787	0.821	0.662	0.755	0.782	+0.124 (<0.001)	+0.034 (0.076)	+0.092 (0.002)	+0.028 (0.607)
Irinotecan	0.652	0.823	0.822	0.680	0.880	0.884	0.684	0.861	0.857	+0.200 (<0.001)	+0.004 (1.000)	+0.177 (<0.001)	-0.004 (1.000)
Nutlin-3a	0.676	0.814	0.832	0.732	0.917	0.924	0.712	0.897	0.901	+0.185 (<0.001)	+0.008 (1.000)	+0.185 (<0.001)	+0.004 (1.000)
Dabrafenib	0.669	0.713	0.723	0.750	0.793	0.796	0.750	0.825	0.814	+0.043 (0.094)	+0.002 (1.000)	+0.075 (0.003)	-0.011 (1.000)
Trametinib	0.715	0.837	0.834	0.736	0.916	0.906	0.717	0.828	0.842	+0.180 (<0.001)	-0.010 (0.241)	+0.112 (<0.001)	+0.014 (0.710)
Lapatinib	0.648	0.690	0.701	0.677	0.774	0.770	0.626	0.744	0.722	+0.097	-0.004	+0.118	-0.022

										(0.002)	(1.000)	(<0.001)	(0.607)
Erlotinib	0.609	0.693	0.714	0.590	0.804	0.795	0.527	0.751	0.749	+0.214 (<0.001)	-0.010 (1.000)	+0.224 (<0.001)	-0.002 (1.000)
Alpelisib	0.642	0.699	0.705	0.677	0.785	0.788	0.646	0.716	0.730	+0.108 (<0.001)	+0.003 (1.000)	+0.070 (0.019)	+0.014 (1.000)

Median: sensitive if LN_IC50 below the drug median (primary definition). Tertile: most sensitive third versus most resistant third, middle third removed. Quartile: most sensitive quarter versus the rest. Differences are paired DeLong differences with Holm adjustment across drugs.

Table S16. Curated drug-specific biomarkers and an OncoTree subtype baseline (re-run cross-validation, median-based labels).

**Drug**	**Lineage AUC**	**Subtype AUC**	**RNA AUC**	**RNA + curated AUC**	**Early AUC**	**Early + curated AUC**	**Subtype minus lineage (Holm p)**	**RNA minus subtype (Holm p)**	**Curated gain, RNA (Holm p)**	**Curated gain, early fusion (Holm p)**
Docetaxel	0.632	0.590	0.738	0.739	0.724	0.723	-0.042 (0.381)	+0.147 (<0.001)	+0.002 (1.000)	-0.001 (1.000)
Paclitaxel	0.610	0.542	0.716	0.742	0.694	0.714	-0.068 (0.054)	+0.174 (<0.001)	+0.025 (0.037)	+0.021 (0.234)
Cisplatin	0.667	0.630	0.792	0.788	0.797	0.792	-0.037 (0.381)	+0.161 (<0.001)	-0.003 (1.000)	-0.005 (1.000)
Gemcitabine	0.618	0.574	0.794	0.792	0.813	0.814	-0.044 (0.188)	+0.221 (<0.001)	-0.002 (1.000)	+0.002 (1.000)
5-Fluorouracil	0.669	0.624	0.740	0.744	0.757	0.767	-0.045 (0.091)	+0.116 (<0.001)	+0.003 (1.000)	+0.010 (0.463)
Irinotecan	0.652	0.653	0.823	0.822	0.822	0.824	+0.002 (0.929)	+0.170 (<0.001)	-0.001 (1.000)	+0.001 (1.000)
Nutlin-3a	0.676	0.661	0.814	0.824	0.832	0.836	-0.015 (0.929)	+0.152 (<0.001)	+0.011 (0.606)	+0.004 (1.000)
Dabrafenib	0.669	0.615	0.713	0.699	0.723	0.733	-0.054 (0.006)	+0.098 (<0.001)	-0.014 (0.255)	+0.010 (0.672)
Trametinib	0.715	0.731	0.837	0.838	0.834	0.838	+0.016 (0.929)	+0.106 (<0.001)	+0.001 (1.000)	+0.003 (1.000)
Lapatinib	0.648	0.597	0.690	0.688	0.701	0.698	-0.051 (0.054)	+0.094 (0.001)	-0.002 (1.000)	-0.004 (1.000)
Erlotinib	0.609	0.566	0.693	0.697	0.714	0.717	-0.043 (0.188)	+0.127 (<0.001)	+0.004 (1.000)	+0.003 (1.000)
Alpelisib	0.642	0.608	0.699	0.701	0.705	0.713	-0.035 (0.381)	+0.091 (<0.001)	+0.002 (1.000)	+0.008 (0.984)

Subtype: indicators for OncoTree primary disease (58 categories) in place of the 26 lineage indicators. Curated: drug-specific genes in Table S20 forced into the expression features (and copy number of the listed amplified drivers into the copy-number block) when the unsupervised filter did not select them.

Table S17a. Leave-one-lineage-out AUC of the expression model with bootstrap 95% CI for every drug-lineage pair.

Drug	Withheld lineage	Cell lines	LOCO AUC	Bootstrap 95% CI
5-Fluorouracil	Bone	18	0.642	0.347 to 0.900
Alpelisib	Bone	18	0.333	0.091 to 0.605
Cisplatin	Bone	18	0.815	0.556 to 0.975
Dabrafenib	Bone	18	0.469	0.195 to 0.762
Docetaxel	Bone	18	0.358	0.111 to 0.653
Erlotinib	Bone	18	0.469	0.188 to 0.753
Gemcitabine	Bone	18	0.556	0.263 to 0.831
Irinotecan	Bone	18	0.691	0.416 to 0.909
Lapatinib	Bone	18	0.716	0.446 to 0.951
Nutlin-3a	Bone	18	0.642	0.338 to 0.893
Paclitaxel	Bone	18	0.691	0.400 to 0.988
Trametinib	Bone	18	0.469	0.185 to 0.762
5-Fluorouracil	Bowel	27	0.522	0.297 to 0.741
Alpelisib	Bowel	27	0.440	0.218 to 0.676
Cisplatin	Bowel	24	0.875	0.708 to 0.993
Dabrafenib	Bowel	26	0.568	0.325 to 0.792
Docetaxel	Bowel	27	0.621	0.390 to 0.833
Erlotinib	Bowel	26	0.462	0.248 to 0.701
Gemcitabine	Bowel	27	0.802	0.611 to 0.959

Irinotecan	Bowel	27	0.846	0.671 to 0.967
Lapatinib	Bowel	27	0.451	0.222 to 0.676
Nutlin-3a	Bowel	27	0.907	0.767 to 1.000
Paclitaxel	Bowel	26	0.586	0.339 to 0.799
Trametinib	Bowel	27	0.555	0.335 to 0.786
5-Fluorouracil	Breast	41	0.683	0.498 to 0.841
Alpelisib	Breast	40	0.598	0.403 to 0.775
Cisplatin	Breast	41	0.610	0.430 to 0.785
Dabrafenib	Breast	40	0.535	0.356 to 0.722
Docetaxel	Breast	41	0.610	0.431 to 0.778
Erlotinib	Breast	40	0.500	0.309 to 0.690
Gemcitabine	Breast	40	0.670	0.484 to 0.836
Irinotecan	Breast	40	0.593	0.414 to 0.771
Lapatinib	Breast	40	0.550	0.363 to 0.732
Nutlin-3a	Breast	41	0.555	0.372 to 0.730
Paclitaxel	Breast	40	0.580	0.389 to 0.765
Trametinib	Breast	40	0.738	0.576 to 0.885
5-Fluorouracil	CNS/Brain	28	0.510	0.287 to 0.734
Alpelisib	CNS/Brain	28	0.617	0.392 to 0.821
Cisplatin	CNS/Brain	22	0.620	0.353 to 0.868
Dabrafenib	CNS/Brain	27	0.566	0.318 to 0.791
Docetaxel	CNS/Brain	28	0.449	0.219 to 0.682
Erlotinib	CNS/Brain	28	0.556	0.328 to 0.789

Gemcitabine	CNS/Brain	28	0.842	0.653 to 0.990
Irinotecan	CNS/Brain	28	0.760	0.547 to 0.924
Lapatinib	CNS/Brain	28	0.515	0.287 to 0.730
Nutlin-3a	CNS/Brain	28	0.418	0.206 to 0.646
Paclitaxel	CNS/Brain	28	0.449	0.226 to 0.679
Trametinib	CNS/Brain	28	0.495	0.262 to 0.724
5-Fluorouracil	Esophagus/Stomach	34	0.602	0.396 to 0.804
Alpelisib	Esophagus/Stomach	34	0.478	0.281 to 0.695
Cisplatin	Esophagus/Stomach	32	0.633	0.417 to 0.826
Dabrafenib	Esophagus/Stomach	34	0.664	0.465 to 0.850
Docetaxel	Esophagus/Stomach	34	0.657	0.460 to 0.852
Erlotinib	Esophagus/Stomach	34	0.453	0.249 to 0.654
Gemcitabine	Esophagus/Stomach	34	0.775	0.600 to 0.919
Irinotecan	Esophagus/Stomach	34	0.574	0.371 to 0.774
Lapatinib	Esophagus/Stomach	34	0.837	0.688 to 0.954
Nutlin-3a	Esophagus/Stomach	34	0.803	0.639 to 0.933
Paclitaxel	Esophagus/Stomach	34	0.529	0.332 to 0.739
Trametinib	Esophagus/Stomach	34	0.571	0.372 to 0.765
5-Fluorouracil	Head and Neck	15	0.446	0.130 to 0.778
Alpelisib	Head and Neck	15	0.393	0.089 to 0.722
Dabrafenib	Head and Neck	15	0.464	0.160 to 0.796
Docetaxel	Head and Neck	15	0.643	0.321 to 0.926
Erlotinib	Head and Neck	15	0.250	0.036 to 0.537

Gemcitabine	Head and Neck	15	0.893	0.630 to 1.000
Irinotecan	Head and Neck	15	0.679	0.321 to 1.000
Lapatinib	Head and Neck	15	0.411	0.091 to 0.750
Nutlin-3a	Head and Neck	15	0.429	0.111 to 0.750
Paclitaxel	Head and Neck	15	0.393	0.093 to 0.714
Trametinib	Head and Neck	15	0.357	0.089 to 0.680
5-Fluorouracil	Kidney	17	0.528	0.250 to 0.800
Alpelisib	Kidney	17	0.417	0.139 to 0.742
Dabrafenib	Kidney	17	0.806	0.542 to 1.000
Docetaxel	Kidney	17	0.917	0.727 to 1.000
Erlotinib	Kidney	17	0.861	0.628 to 1.000
Gemcitabine	Kidney	17	0.694	0.386 to 0.939
Irinotecan	Kidney	17	0.750	0.472 to 0.985
Lapatinib	Kidney	17	0.431	0.143 to 0.736
Nutlin-3a	Kidney	17	0.750	0.444 to 0.972
Paclitaxel	Kidney	17	0.569	0.258 to 0.886
Trametinib	Kidney	17	0.528	0.229 to 0.833
5-Fluorouracil	Lung	99	0.553	0.436 to 0.676
Alpelisib	Lung	99	0.627	0.516 to 0.732
Cisplatin	Lung	72	0.740	0.618 to 0.861
Dabrafenib	Lung	97	0.611	0.492 to 0.725
Docetaxel	Lung	99	0.674	0.564 to 0.772
Erlotinib	Lung	98	0.579	0.459 to 0.690

Gemcitabine	Lung	98	0.611	0.491 to 0.727
Irinotecan	Lung	99	0.688	0.582 to 0.782
Lapatinib	Lung	99	0.587	0.472 to 0.695
Nutlin-3a	Lung	99	0.590	0.473 to 0.701
Paclitaxel	Lung	98	0.638	0.524 to 0.745
Trametinib	Lung	99	0.548	0.434 to 0.657
5-Fluorouracil	Ovary/Fallopian Tube	24	0.535	0.279 to 0.783
Alpelisib	Ovary/Fallopian Tube	24	0.382	0.168 to 0.641
Cisplatin	Ovary/Fallopian Tube	19	0.922	0.769 to 1.000
Dabrafenib	Ovary/Fallopian Tube	24	0.354	0.139 to 0.600
Docetaxel	Ovary/Fallopian Tube	24	0.729	0.493 to 0.933
Erlotinib	Ovary/Fallopian Tube	24	0.646	0.396 to 0.859
Gemcitabine	Ovary/Fallopian Tube	24	0.736	0.507 to 0.944
Irinotecan	Ovary/Fallopian Tube	24	0.889	0.708 to 1.000
Lapatinib	Ovary/Fallopian Tube	24	0.639	0.393 to 0.871
Nutlin-3a	Ovary/Fallopian Tube	24	0.757	0.529 to 0.943
Paclitaxel	Ovary/Fallopian Tube	24	0.576	0.328 to 0.807
Trametinib	Ovary/Fallopian Tube	24	0.493	0.244 to 0.741
5-Fluorouracil	Pancreas	23	0.523	0.273 to 0.773
Alpelisib	Pancreas	23	0.439	0.197 to 0.697
Cisplatin	Pancreas	22	0.860	0.670 to 0.990
Dabrafenib	Pancreas	23	0.606	0.356 to 0.841
Docetaxel	Pancreas	23	0.818	0.611 to 0.962

Erlotinib	Pancreas	23	0.462	0.227 to 0.720
Gemcitabine	Pancreas	23	0.811	0.598 to 0.991
Irinotecan	Pancreas	23	0.705	0.444 to 0.937
Lapatinib	Pancreas	23	0.409	0.167 to 0.674
Nutlin-3a	Pancreas	23	0.629	0.386 to 0.871
Paclitaxel	Pancreas	23	0.773	0.546 to 0.946
Trametinib	Pancreas	23	0.606	0.362 to 0.849
5-Fluorouracil	Skin	28	0.821	0.630 to 0.964
Alpelisib	Skin	28	0.485	0.262 to 0.714
Cisplatin	Skin	19	0.556	0.273 to 0.833
Dabrafenib	Skin	27	0.495	0.261 to 0.725
Docetaxel	Skin	28	0.770	0.577 to 0.934
Erlotinib	Skin	28	0.495	0.257 to 0.713
Gemcitabine	Skin	28	0.638	0.415 to 0.836
Irinotecan	Skin	28	0.648	0.415 to 0.848
Lapatinib	Skin	28	0.546	0.316 to 0.774
Nutlin-3a	Skin	28	0.327	0.122 to 0.546
Paclitaxel	Skin	28	0.556	0.333 to 0.765
Trametinib	Skin	28	0.449	0.221 to 0.677

Table S17b. LOCO uncertainty summarised by withheld lineage (expression model).

Lineage	Drug pairs	Cell lines	Median LOCO AUC	IQR	Median CI width	CI above 0.5	CI below 0.5

Lung	12	72-99	0.611	0.585-0.647	0.226	5	0
Breast	12	40-41	0.595	0.554-0.625	0.357	1	0
Esophagus/Stomach	12	32-34	0.617	0.561-0.692	0.398	3	0
CNS/Brain	12	22-28	0.536	0.483-0.618	0.450	2	0
Skin	12	19-28	0.551	0.492-0.640	0.443	2	0
Bowel	12	24-27	0.577	0.507-0.813	0.448	4	0
Ovary/Fallopian Tube	12	19-24	0.642	0.524-0.741	0.462	4	0
Pancreas	12	22-23	0.617	0.508-0.782	0.486	4	0
Bone	12	18-18	0.599	0.469-0.691	0.554	1	0
Kidney	11	17-17	0.694	0.528-0.778	0.550	3	0
Head and Neck	11	15-15	0.429	0.393-0.554	0.633	1	0

CI above (below) 0.5: number of drug-lineage pairs whose bootstrap 95% CI lies entirely above (below) 0.5.

Table S17c. Effect of the minimum lineage size on the mixed-lineage versus LOCO comparison.

Model	Minimum cell lines	Pairs	Lineages	Median gap (mixed minus LOCO)	Mixed higher	Wilcoxon p	LOCO AUC < 0.5
RNA	15	130	11	+0.030	85/130	<0.001	35
RNA	20	94	8	+0.031	63/94	0.001	20
RNA	25	69	6	+0.027	45/69	0.011	14
RNA	30	36	3	+0.011	21/36	0.355	2
Early	15	130	11	+0.029	80/130	0.004	26
Early	20	94	8	+0.032	59/94	0.006	16
Early	25	69	6	+0.028	42/69	0.084	12

Early	30	36	3	+0.012	20/36	0.265	1

Table S18. External validation on PRISM rebuilt from the raw secondary screen, with the late-fusion and curated-biomarker models and three definitions of PRISM sensitivity.

Drug	External n	Cohort median: fraction sensitive	Cohort median: Tissue	Cohort median: RNA	Cohort median: Early	Cohort median: Late	Cohort median: RNA+curated	Fixed (overlap median): fraction sensitive	Fixed (overlap median): Tissue	Fixed (overlap median): RNA	Fixed (overlap median): Early	Fixed (overlap median): Late	Fixed (overlap median): RNA+curated	PRISM-wide median: fraction sensitive	PRISM-wide median: Tissue	PRISM-wide median: RNA	PRISM-wide median: Early	PRISM-wide median: Late	PRISM-wide median: RNA+curated	RNA vs PRISM AUC (rho, threshold-free)
Docetaxel	158	0.50	0.530	0.511	0.537	0.526	0.530	0.46	0.529	0.524	0.551	0.546	0.538	0.46	0.528	0.517	0.545	0.538	0.533	0.04
Paclitaxel	159	0.50	0.514	0.543	0.535	0.545	0.541	0.40	0.487	0.559	0.552	0.564	0.576	0.43	0.492	0.537	0.543	0.543	0.557	0.06
Cisplatin	119	0.50	0.504	0.473	0.449	0.455	0.470	0.48	0.512	0.497	0.469	0.463	0.493	0.49	0.496	0.486	0.461	0.456	0.484	0.00
Gemcitabine	77	0.49	0.616	0.706	0.632	0.660	0.694	0.53	0.620	0.675	0.608	0.635	0.650	0.53	0.620	0.675	0.608	0.635	0.650	0.37
5-Fluorouracil	89	0.49	0.553	0.525	0.511	0.524	0.555	0.58	0.573	0.570	0.548	0.569	0.583	0.58	0.573	0.570	0.548	0.569	0.583	0.07
Irinotecan	162	0.50	0.613	0.715	0.707	0.711	0.709	0.60	0.597	0.706	0.697	0.702	0.704	0.58	0.601	0.712	0.692	0.703	0.705	0.45
Nutlin-3a	89	0.49	0.581	0.730	0.760	0.753	0.740	0.35	0.574	0.740	0.782	0.777	0.759	0.42	0.569	0.729	0.757	0.751	0.741	0.45
Dabrafenib	153	0.50	0.574	0.618	0.644	0.618	0.627	0.56	0.555	0.626	0.647	0.629	0.640	0.54	0.548	0.620	0.644	0.622	0.629	0.31
Trameti	163	0.50	0.610	0.743	0.721	0.756	0.760	0.53	0.619	0.748	0.731	0.757	0.764	0.52	0.616	0.736	0.721	0.750	0.754	0.50

nib																			
Lapatinib	152	0.50	0.620	0.562	0.545	0.572	0.539	0.52	0.613	0.568	0.545	0.574	0.544	0.48	0.607	0.590	0.579	0.601	0.567	0.19
Erlotinib	163	0.50	0.487	0.598	0.635	0.605	0.608	0.44	0.505	0.617	0.637	0.620	0.623	0.45	0.487	0.607	0.638	0.616	0.616	0.18
Alpelisib	157	0.50	0.563	0.655	0.668	0.663	0.645	0.50	0.561	0.662	0.667	0.668	0.648	0.50	0.563	0.655	0.668	0.663	0.645	0.39

Cohort median: median PRISM AUC of the evaluation cohort (published definition). Fixed (overlap median): median PRISM AUC of cell lines screened in both GDSC2 and PRISM, none of which is in the evaluation cohort. PRISM-wide median: median over every STR-passed PRISM cell line screened with the drug. Models were trained on the full GDSC2 cohort with a fixed seed that differs from the published run; bootstrap CIs are in the Excel workbook.

Table S19. Robustness of the association between GDSC2-PRISM concordance and external AUC across the 12 drugs.

Model	Spearman rho	p	Bootstrap 95% CI (drugs)	Leave-one-drug-out rho range	Largest leave-one-out p	rho internal vs external AUC	rho internal AUC vs concordance	Partial rho (adjusted for internal AUC)	Partial p
RNA	0.72	0.008	0.14 to 1.00	0.64 to 0.86	0.035	0.50	0.50	0.63	0.039
Early	0.66	0.018	0.00 to 0.96	0.56 to 0.82	0.071	0.61	0.55	0.50	0.121

Bootstrap: 10,000 resamples of drugs. Partial correlation: Spearman correlation between concordance and external AUC after adjustment for internal cross-validated AUC of the same model.

Table S20. Curated drug-specific biomarkers used in the sensitivity analysis.

Drug	Curated expression genes	Curated copy-number genes
Docetaxel	ABCB1, ABCG2, ABCC1, TUBB3, MAPT, BCL2L1	none
Paclitaxel	ABCB1, ABCG2, ABCC1, TUBB3, MAPT, BCL2L1	none
Cisplatin	SLFN11, ERCC1, GSTP1, ABCC2, MLH1, ATP7B	none
Gemcitabine	SLFN11, DCK, RRM1, RRM2, SLC29A1, CDA	none
5-Fluorouracil	SLFN11, TYMS, DPYD, TYMP, UMPS, MTHFR	none

Irinotecan	SLFN11, TOP1, UGT1A1, ABCG2, CES2, ABCB1	none
Nutlin-3a	MDM2, CDKN1A, TP53, MDM4, BAX, ZMAT3	MDM2, MDM4
Dabrafenib	BRAF, DUSP6, ETV4, ETV5, SPRY2, SPRY4	BRAF
Trametinib	DUSP6, ETV4, ETV5, SPRY2, SPRY4, FOSL1	BRAF, KRAS
Lapatinib	ERBB2, EGFR, ERBB3, GRB7, ESR1, PTEN	ERBB2, EGFR
Erlotinib	EGFR, ERBB3, AREG, EREG, ERBB2, MET	EGFR, ERBB2, MET
Alpelisib	PIK3CA, PTEN, ESR1, ERBB2, ERBB3, PIK3R1	PIK3CA, ERBB2

Table S21 (drug-cell line inclusion table with LN_IC50, lineage, OncoTree primary disease and class label for every analysed pair) and Table S22 (features selected for each drug when the model is trained on the full cohort, with curated additions flagged) are provided in the Excel workbook and as CSV files.
